# Occurrence and Characterization of *mcr-1*-Positive *Escherichia coli* Isolated From Food-Producing Animals in Poland, 2011–2016

**DOI:** 10.3389/fmicb.2019.01753

**Published:** 2019-08-08

**Authors:** Magdalena Zając, Paweł Sztromwasser, Valeria Bortolaia, Pimlapas Leekitcharoenphon, Lina M. Cavaco, Anna Ziȩtek-Barszcz, Rene S. Hendriksen, Dariusz Wasyl

**Affiliations:** ^1^Department of Microbiology, National Veterinary Research Institute, Puławy, Poland; ^2^Department of Omics Analyses, National Veterinary Research Institute, Puławy, Poland; ^3^Research Group for Genomic Epidemiology, European Union Reference Laboratory for Antimicrobial Resistance, WHO Collaborating Centre for Antimicrobial Resistance in Foodborne Pathogens and Genomics, National Food Institute, Technical University of Denmark, Lyngby, Denmark; ^4^Statens Serum Institute, Copenhagen University, Copenhagen, Denmark; ^5^Department of Epidemiology, National Veterinary Research Institute, Puławy, Poland

**Keywords:** WGS, *mcr-1*, colistin resistance, aEPEC, food animal, IncX4, IncHI2

## Abstract

The emergence of plasmid-mediated colistin resistance (*mcr* genes) threatens the effectiveness of polymyxins, which are last-resort drugs to treat infections by multidrug- and carbapenem-resistant Gram-negative bacteria. Based on the occurrence of colistin resistance the aims of the study were to determine possible resistance mechanisms and then characterize the *mcr*-positive *Escherichia coli*. The research used material from the Polish national and EU harmonized antimicrobial resistance (AMR) monitoring programs. A total of 5,878 commensal *E. coli* from fecal samples of turkeys, chickens, pigs, and cattle collected in 2011–2016 were screened by minimum inhibitory concentration (MIC) determination for the presence of resistance to colistin (R) defined as *R* > 2 mg/L. Strains with MIC = 2 mg/L isolated in 2014–2016 were also included. A total of 128 isolates were obtained, and most (66.3%) had colistin MIC of 2 mg/L. PCR revealed *mcr-1* in 80 (62.5%) isolates recovered from 61 turkeys, 11 broilers, 2 laying hens, 1 pig, and 1 bovine. No other *mcr*-type genes (including *mcr-2* to -*5*) were detected. Whole-genome sequencing (WGS) of the *mcr-1*–positive isolates showed high diversity in the multi-locus sequence types (MLST) of *E. coli*, plasmid replicons, and AMR and virulence genes. Generally *mcr-1.1* was detected on the same contig as the IncX4 (76.3%) and IncHI2 (6.3%) replicons. One isolate harbored *mcr-1*.*1* on the chromosome. Various extended-spectrum beta-lactamase (*bla*_*SHV*–12_, *bla*_*CTX*–*M*–1_, *bla*_*CTX*–*M*–15_, *bla*_*TEM*–30_, *bla*_*TEM*–52_, and *bla*_*TEM*–135_) and quinolone resistance genes (*qnrS1*, *qnrB19*, and chromosomal *gyrA, parC*, and *parE* mutations) were present in the *mcr-1*.*1*–positive *E. coli*. A total of 49 sequence types (ST) were identified, ST354, ST359, ST48, and ST617 predominating. One isolate, identified as ST189, belonged to atypical enteropathogenic *E. coli.* Our findings show that *mcr-1*.*1* has spread widely among production animals in Poland, particularly in turkeys and appears to be transferable mainly by IncX4 and IncHI2 plasmids spread across diverse *E. coli* lineages. Interestingly, most of these *mcr-1*–positive *E. coli* would remain undetected using phenotypic methods with the current epidemiological cut-off value (ECOFF). The appearance and spread of *mcr-1* among various animals, but notably in turkeys, might be considered a food chain, and public health hazard.

## Introduction

The worldwide increase in the occurrence of antimicrobial resistance (AMR) and prevalence of multidrug-resistant (MDR) Gram-negative *Enterobacteriaceae* challenge our ability to treat infections in humans and animals, thus resulting in a renewed interest in old drugs such as polymyxins. Colistin (polymyxin E), which has been used in veterinary practice for decades mainly for treating Gram-negative bacteria infections of the gastrointestinal tract in pigs, poultry and cattle, is nowadays considered a last-resort drug to treat human infections by multidrug-, and carbapenem-resistant Gram-negative bacteria. Together with third, fourth, and fifth generation cephalosporins, glycopeptides, quinolones, and macrolides, polymyxins are among the critically important antimicrobials (CIA) for human medicine (World Human Organization [WHO], 2017) and should be mainly used for treating the severest human infections to preserve their effectiveness. Antimicrobials are used in hospitals and care facilities as well as in veterinary clinics and on farms. Extensive use of antimicrobials is recognized as the most important factor selecting for AMR in bacteria (Centers for Disease Control and Prevention [CDC], 2013). According to the European Surveillance of Veterinary Antimicrobial Consumption (ESVAC) report, sales of veterinary antimicrobial agents in 2016 varied from 0.7 to 2,726.5 tons in the 30 participating countries (European Medicine Agency [EMA] and European Surveillance of Veterinary Antimicrobial Consumption [ESVAC], 2018). Notably, polymyxins were the fifth most sold group of antimicrobials in 2015–2016 (European Medicine Agency [EMA] and European Surveillance of Veterinary Antimicrobial Consumption [ESVAC], 2017, 2018). In Poland, colistin sales increased by 35% from 2011 to 2016, reaching their highest value of 5.94 mg per population correction unit (PCU) in 2015 and exceeding the recommended maximum sale target of 5 mg/PCU for this antimicrobial (European Medicine Agency [EMA] and European Surveillance of Veterinary Antimicrobial Consumption [ESVAC], 2016, 2017, 2018). Currently, there are 26 veterinary medicinal products containing colistin (*Colistini sulfas* or colistinum) registered in Poland as powders for oral solution, with six registered only in 2017^1^.

In *Enterobacteriaceae*, resistance to polymyxines was theorized to be regulated by the two-component systems PhoP/PhoQ and PmrA/PmrB involved in LPS modifications (Olaitan et al., 2014). The emergence and spread of plasmid-mediated colistin resistance (the *mcr-1* gene), first described in China in 2015 (Liu et al., 2016), and poses a threat to the effectiveness of colistin. The *mcr-1* gene has been detected in several bacterial species (Li et al., 2017; Tian et al., 2017; Torpdahl et al., 2017) in association with different plasmid types such as IncI2, IncHI2, IncP, IncFIP, and IncX4 and also inserted into the bacterial chromosome (Liu et al., 2016; Zurfluh et al., 2016; Hadjadj et al., 2017; Sun et al., 2018). New *mcr* genes and their variants have also been identified: *mcr*-*2* (Xavier et al., 2016)*, mcr-3* (Yin et al., 2017)*, mcr-4* (Carattoli et al., 2017)*, mcr-*5 (Borowiak et al., 2017), *mcr-6* (Abuoun et al., 2017), *mcr-7* (Yang et al., 2018), *mcr-8* (Wang et al., 2018), and *mcr-9* (Carroll et al., 2019).

Little is known about the prevalence of colistin resistance and the occurrence of *mcr* genes in livestock in Poland. In 2015, a single case of *mcr-1*–positive *Escherichia coli* was described from a human patient with a urinary tract infection (Izdebski et al., 2016). This might be the first evidence from Poland that *mcr*-mediated colistin resistance from animals has spread to humans, which would validate concerns over foodborne transfer of colistin-resistant bacteria to humans (Grami et al., 2016). Based on investigation of the occurrence of colistin resistance among *E. coli* isolated from food-producing animals in Poland over a 6-year period, the aim of the study was to determine the resistance mechanisms among the colistin-resistant isolates. Whole genome sequence analysis of the *mcr-1*–positive *E. coli* strains was made to elucidate the pathways of dissemination of *mcr-1* in food-producing animals in Poland and highlight possible animal and public health threats.

## Materials and Methods

### Bacterial Isolates

A total of 5,878 commensal *E. coli* isolates were obtained from individual fecal samples collected from turkeys, chickens, pigs and cattle in 2011–2016, and tested for antimicrobial susceptibility by minimum inhibitory concentration (MIC) determination (Sensititre, TREK Diagnostic; EUMVS2 and EUVSEC plates). The isolates were screened to confirm the presence of microbiological resistance (*R*) to colistin (*R* > 2 mg/L). Additionally, available isolates with MIC = 2 mg/L (wild-type isolates) from 2014 to 2016 were included in the study because they represented colistin MIC values one dilution step from those considered as non-wild type (NWT). Isolates were collected as part of the multiannual national program (2011–2016) and the EU harmonized AMR monitoring program carried out in 2014–2016 (Decision 2013/652/EU). Those programs are based on isolation of commensal *E. coli* from the cecal content of samples collected from random animals at slaughter. The sampling was carried out by veterinary officers on a by-slaughterhouse basis proportionally to the annual capacity of the slaughterhouse and at intervals distributed over the 6-year period. The antimicrobial susceptibility testing (AST) for ampicillin, azithromycin, cefotaxime, ceftazidime, chloramphenicol, ciprofloxacin, gentamicin, colistin, nalidixic acid, meropenem, sulfamethoxazole, tetracycline, tigecycline, and trimethoprim was interpreted according to the European Committee on Antimicrobial Susceptibility Testing (EUCAST) criteria describing epidemiological cut-off values (ECOFFs) for antimicrobials. The selected isolates were subjected to PCR targeting the *mcr-1* and *mcr*-2 genes (Cavaco et al., 2016). Subsequently, resistant isolates were whole-genome sequenced (WGS) as detailed below. PCR-negative strains were re-tested phenotypically to confirm the MIC to colistin and screened for the presence of *mcr-1*, -*2, -3, -4*, and -*5* using PCR (Rebelo et al., 2018).

### Whole Genome Sequencing

DNA from bacterial cells of the 80 *mcr-1*–positive isolates was extracted from nutrient agar plate cultures using a Genomic Mini Kit (A&A Biotechnology) following the manufacturer’s recommendations. Sequencing libraries were prepared with the Nextera XT DNA Sample Preparation Kit (Illumina) according to the manufacturer’s protocol. Sequencing of the strains was performed using Illumina MiSeq 2 bp × 250 bp and 2 bp × 300 bp reads or Illumina HiSeq 2 bp × 150 bp reads, generating on average 398 Mb per sample (176–673 Mb), which corresponds to average coverage of 80× (35–135×) in a 5 Mb genome. The raw reads were processed using bbmerge v36.62 (Bushnell, 2018) to merge overlapping reads and Trimmomatic v0.36 (Bolger et al., 2014) to trim adapters and low quality reads. Merged reads and trimmed unmerged pairs were used to generate assembly contigs and scaffolds using SPAdes 3.9.0 (Bankevich et al., 2012). The mean N50 of assemblies was 178 kb (77–433 kb) and the average number of contigs longer than 1 kb was 102 (40–364). Six isolates where the *mcr* gene was not located on the same contig as a plasmid replicon were subjected to additional Pacific Biosciences long-read sequencing, three samples per SMRTcell. The raw PacBio reads were de-multiplexed to subreads using *lima* 1.0.0 (Pacific Biosciences) (Topfer, 2018) yielding on average 225 Mb per sample (72–390 Mb), which translates to average 45× coverage (14.4–78×) of a 5 Mbps genome. The mean subread length was 3,555 bp (3,183–3,929 bp) and mean basepair quality 13.1 (12.95–13.22). Subreads were used in a hybrid SPAdes assembly together with raw short Illumina reads. Assembly analysis with QUAST 4.5 (Gurevich et al., 2013) reported 8–13 contigs longer than 10 kb per sample and 2.1 Mb average N50 (0.91–3.9 Mb). The DNA sequences (reads) from the isolates were deposited in the European Nucleotide Archive (ENA) under project number PRJEB23993. Specific sequence numbers are included in Supplementary Table S1. *E. coli* strains which codes start from “U” were gathered within antimicrobial resistance monitoring according to 2013/652/EC and they are included in the annual EFSA/ECDC reports.

### Bioinformatic Data Analysis

Sequences were analyzed for the presence of AMR genes, virulence genes and plasmid replicons by using the Center for Genomic Epidemiology (CGE)^2^ ResFinder 3.1.0 (with database updated on September 10, 2018) (Zankari et al., 2012), VirulenceFinder 1.5 (February 18, 2016) (Joensen et al., 2014), PlasmidFinder 1.3 (December 15, 2017) (Carattoli et al., 2014), and pMLST v1.4 (December 15, 2017) (Carattoli et al., 2014) web-based tools for typing of IncHI2 plasmids. The criteria for these tools were: 90% threshold for identity with the reference and minimum 60% coverage of the gene length. Multi-locus sequence typing (MLST) of strains was performed using MLST 1.8 (Carattoli et al., 2014). The phylogenetic tree of 80 isolates was constructed by complete linkage clustering using a sequence similarity distance matrix. The distance matrix was generated by global pairwise MUMmer 3.23 (Kurtz et al., 2004) alignments between samples’ scaffolds, automated by CONCOCT 0.4.0 (Alneberg et al., 2014). A phylogenetic tree of IncX4 plasmids was created in a similar way, using contigs carrying the IncX4 replicon and the *mcr-1* gene. The *mcr-1* carrying contigs were identified using BLAST (Altschul et al., 1990) and *mcr-1* sequence AKF16168.1. The iTol web-based tool (Letunic and Bork, 2016) was used to visualize the trees.

## Results

### Occurrence of Colistin Resistance and *mcr-1*

**FIGURE 1:**
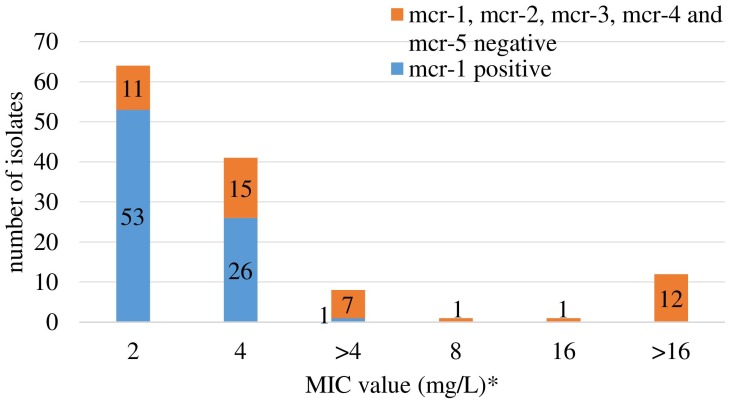
Colistin MIC distribution and occurrence of the *mcr-1* and *mcr-2* gene among 128 *Escherichia coli* selected based on colistin MIC > 2 mg/L (2011–2013) and ≥2 mg/L (2014–2016). ^∗^ only isolates with MIC_*colistin*_ ≥ 2 mg/L were tested for *mcr-1* and *mcr-2* genes. Different MIC values (>4 and 8, 16, and >16) are result of changed MIC panel in plates. EUMVS2 plate (2–4 mg/L) was used in 2011–2013, EUVSEC plate (1–16 mg/L) in 2014–2016.

**FIGURE 2:**
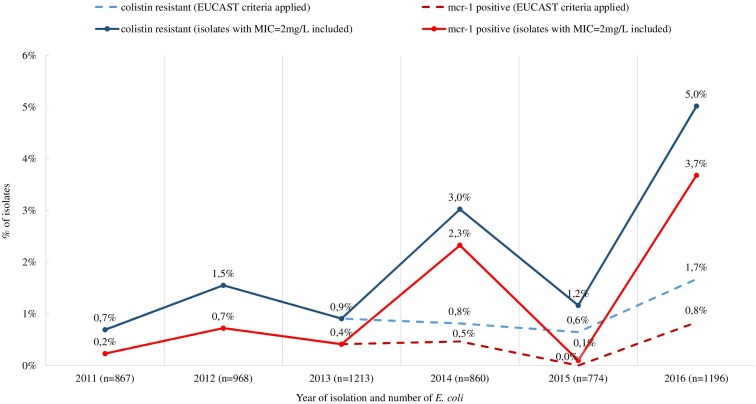
Occurrence of isolates meeting the selection criteria (MIC > 2 mg/L for isolates in 2011–2013 and MIC ≥ 2 mg/L for isolates in 2014–2016) and *mcr-1* positive commensal and ESBL/ampC producing *E. coli* from all tested sources (turkeys, chickens, pigs, and cattle), 2011–2016. The occurrence of colistin resistant and *mcr-1* positive isolates when using exclusively EUCAST ECOFF was included (dashed line).

Retrospective analysis of MIC data revealed a total of 128 (2.2%) out of 5,878 commensal *E. coli* fulfilling the selection criteria with colistin MICs ranging from 2 to 16 mg/L (Figure 1). They originated mostly from turkeys (63%) and chickens (23%). A slight temporal increase of microbiological resistance to colistin from very low to low (0.7–1.7%) was observed when considering all *E. coli* isolates detected in samples from 2011 to 2016 taken from Polish food-producing animals irrespective of their origin (Figure 2).

**FIGURE 3:**
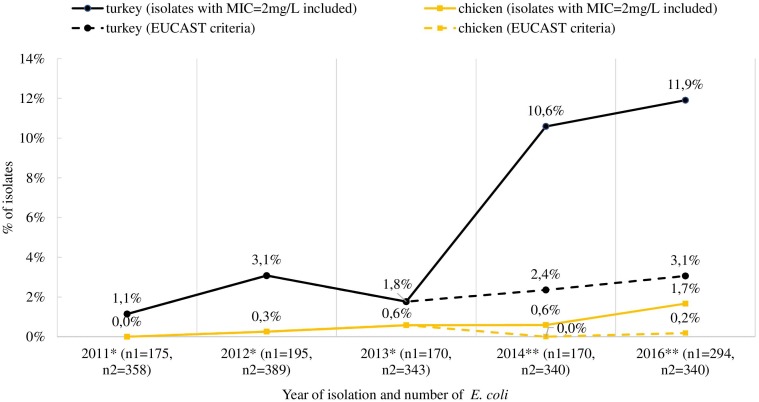
Occurrence of *mcr-1*-positive *E. coli* in Poland isolated from turkey and chicken fecal samples. ^∗^ isolates collected in multiannual governmental programs (according to of the Council of Ministers Decisions), ^∗∗^ isolates deriving from official monitoring according to Decision 2013/652/EC and thus not encompassing samples from turkeys and broilers in 2015; n1 and n2 indicate the total number of isolates tested for MIC determination from turkeys and chickens (both broiler and laying hens), respectively. The occurrence of *mcr-1* positive strains when using exclusively EUCAST ECOFF during selection of isolates was also included (dashed line).

The *mcr-1* gene was detected in 80 (62.5%) out of the selected 128 isolates, deriving from 76 fecal samples recovered from turkeys (*n* = 61), broilers (*n* = 11), laying hens (*n* = 2), pigs (*n* = 1), and cattle (*n* = 1). Most of the *mcr-1*–positive *E. coli* originated from individual samples, but in four samples from turkeys (*n* = 3) and broilers (*n* = 1), two different isolates per sample were identified (Supplementary Table S1). An increase in occurrence of the *mcr-1*–positive *E. coli* was noted in turkey and chicken samples, respectively from 1.1 and 0.0% in 2011 to 11.6 and 1.7% in 2016 (Figure 3). The CGE ResFinder tool confirmed the presence of the *mcr-1*.*1* gene in all PCR-confirmed isolates. No *mcr*-*2, mcr-3, mcr-4*, or *mcr-5* was identified either from PCR or the genome analysis in *mcr-1*–positive isolates. Noteworthily, the *mcr-1*.*1* was mostly found (*n* = 53; 66.3%) in isolates with colistin MIC = 2 mg/L which is the EUCAST ECOFF and regarded as that of the wild-type population. Most of these (*n* = 41; 77.4%) were sampled from turkeys. Additionally, a mutation in the chromosomal *pmrB* gene (Val161→Gly) was detected in one *mcr-1*.*1*–positive isolate with MIC = 2 mg/L.

**FIGURE 4:**
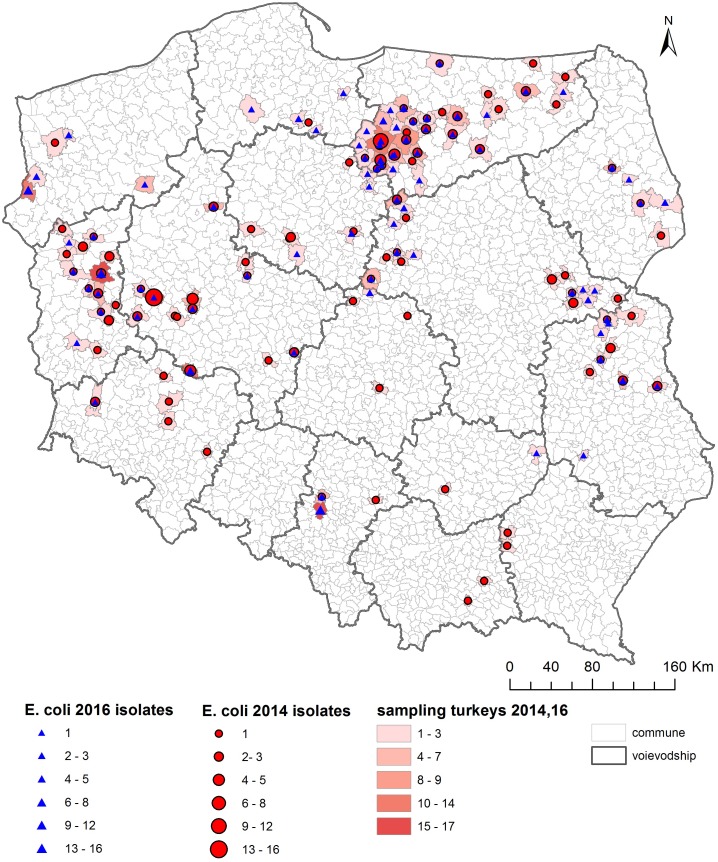
Geographical distribution (commune level) of turkey sampling. Single turkey caeca was collected at slaughter and farm of origin was retrieved for verification of randomization of samples used for isolation of indicator *E. coli* in 2014 and 2016.

**FIGURE 5:**
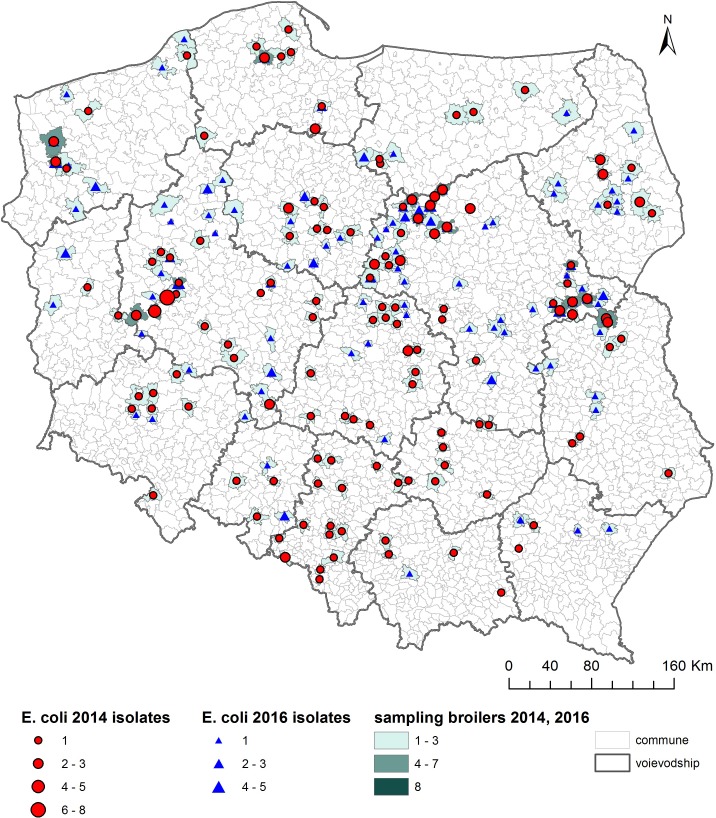
Geographical distribution (commune level) of broiler sampling. Single broiler caeca was collected at slaughter and farm of origin was retrieved for verification of randomization of samples used for isolation of indicator *E. coli* in 2014 and 2016.

**FIGURE 6:**
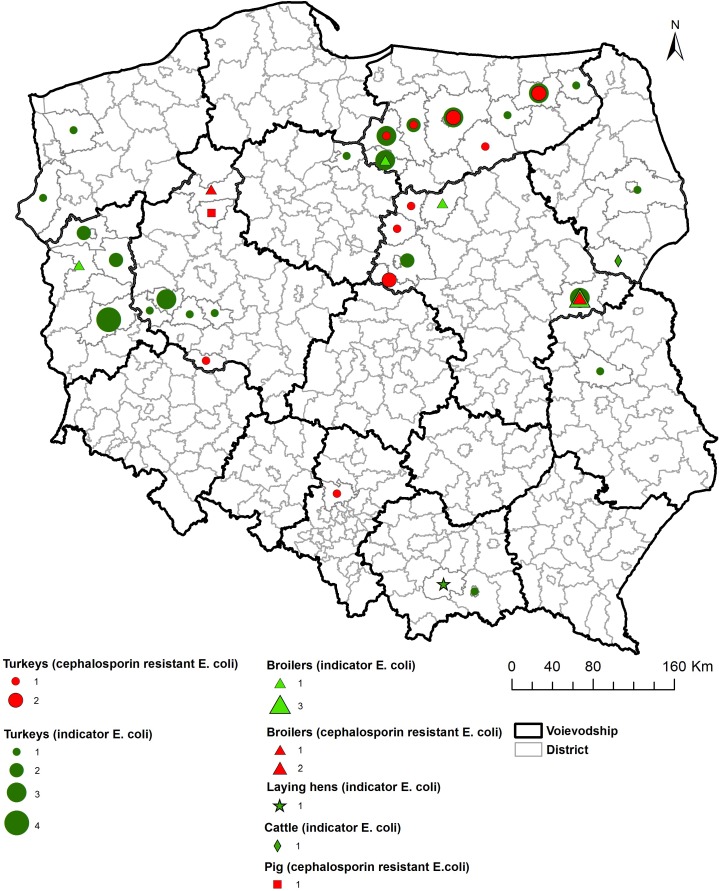
Geographical distribution (district level) of *mcr-1* positive *E. coli* isolation sites.

As shown on the maps of the farm locations from which *mcr-1*–positive *E. coli* was isolated, the colonized farms were distributed over the country with no specific regional trend (Figure 4–6).

An MIC ≥ 2 mg/L for colistin could not be confirmed in any of the re-tested 48 isolates initially suspected but found negative for *mcr-1* and *mcr*-*2*, and none of the *mcr-1*, -*2*, -*3*, -*4*, or -*5* genes were identified by PCR. They were not investigated further as we considered them either false positives in the initial testing, or to have eventually lost the mechanisms over prolonged storage or handling.

### Phylogeny and Epidemiology

**FIGURE 7:**
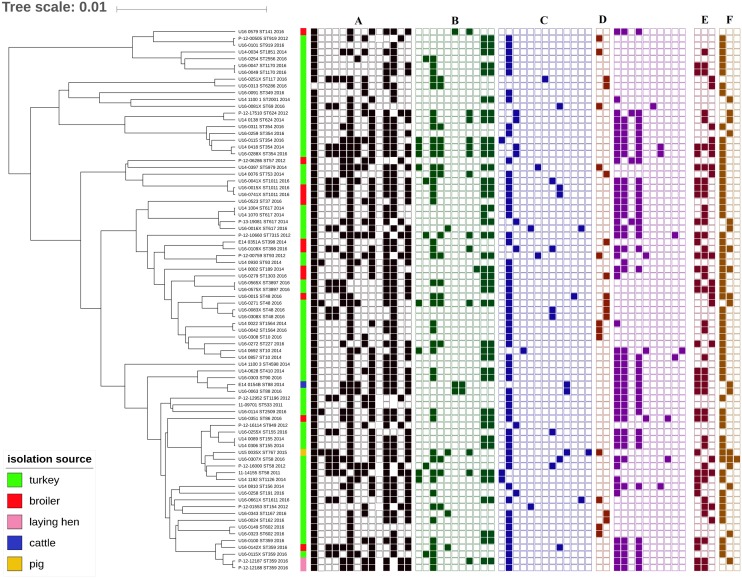
Phylogeny of *mcr-1*-positive *E. coli* (sequence types, year and source of isolation, and map of phenotypic resistance and resistance genes). Full and empty square mean presence and absence of antimicrobial resistance (AMR) gene, respectively, whereas empty space means non-defined or not tested. **(A)** Resistance profiles (black): ampicillin, azithromycin, cefuroxime, ceftazidime, chloramphenicol, ciprofloxacin, gentamicin, colistin, nalidixic acid, meropenem, sulphonamides, tetracycline, tigecycline, and trimethoprim (Sensititre EUSVEC MIC panel). **(B)** Aminoglycoside resistance genes (green): *aac*(3)-IIa, *aac*(3)-IId, *aad*A1, *aad*A2, *aad*A5, *aad*A24, *aad*B, *aph*(3′)-Ia, *aph*(3′)-Ic, *aph*(3″)-Ib, and *aph(6)*-Id. **(C)** Beta-lactam resistance genes (blue): *bla*_*TEM*–1A_, *bla*_*TEM*–1B_, *bla*_*TEM*–1C_, *bla*_*TEM*–30_, *bla*_*TEM*–135_, *bla*_*TEM*–52*C*_, *bla*_*CMY*–2_, *bla*_*SHV*–12_, *bla*_OXA-1_,*bla*_CARB-2_, and *bla*_*CTX*–*M*–1_. **(D)** Quinolone resistance genes: plasmid-mediated quinolone resistance (PMQR, brown): *qnrS1*, *qnrB19*; mutations in quinolone resistance determining regions (QRDR, violet): *gyrA* S83L, *gyrA* D87N, *gyrA* D87Y, *parC* S80I, *parC* S80R, *parC* S57T, *parC* E84G, *parC* E84K, *par*E L416F, and *par*E L460D. **(E)** Sulphonamide resistance genes (dark brown): *sul*1, *sul*2, and *sul*3. **(F)** Tetracycline resistance genes (light brown): *tet*(A), *tet*(B), and *tet*(M).

The MLST revealed 49 ST among the sequenced isolates. In 64 *E. coli* from turkeys, 41 STs were identified, as were 10 in 14 chicken isolates. The most common types were ST354 and ST359, which were observed in five isolates each, ST48 and ST617 which were identified in four isolates each, and ST10, ST58, ST155, and ST1011 which were represented by three isolates each. Single isolates represented 32 ST (Figure 7).

The analysis showed high heterogeneity of *mcr-1*–positive *E. coli* independent of source and year of isolation. Isolates deriving from animals from 27 farms and slaughtered in 27 slaughterhouses (Supplementary Table S2) were clustered according to their ST. The majority of isolates belonging to the most numerous STs (i.e., ST48, ST88, ST359, and ST1011) derived from different animal species (Figure 7) slaughtered in different slaughterhouses (Supplementary Table S1) and originating from different farms or flocks (data not shown). In cases where the same ST was found in animals from the same slaughterhouse and/or farm, the *mcr* localization and plasmid profile were often different, as for example with ST354 observed exclusively in turkeys where two isolates deriving from the same farm but slaughtered in different places had *mcr* localized on IncX4 (U16_0311), and chromosome (U16_0259) (Supplementary Table S1). In a few cases, the same ST (i.e., ST354, ST359, ST919, and ST1564) was present among strains isolated in different years.

### Phenotypic and Genetic Traits of Microbiological Resistance to Additional Antimicrobials

The *mcr-1*–positive strains showed resistance to at least two and up to seven different classes of antimicrobials and had different resistance gene contents. Seventy-eight (97.5%) *mcr-1*-positive *E. coli* were classified as MDR isolates. Seventy-nine (98.8%) were resistant to ampicillin and 22 (27.5%) to cefotaxime and ceftazidime. Resistance to ciprofloxacin was confirmed in 70 (87.5%), to tetracycline in 61 (76.3%), to nalidixic acid in 50 (62.5%), to chloramphenicol in 27 (33.8%), to gentamicin in 16 (20.0%), and to tigecycline in 12 (15.0%). Four of the isolates had an azithromycin MIC ≥ 16 mg/L, which can be interpreted as resistance according to the tentative ECOFF for this antimicrobial. The strains were susceptible to meropenem and presented no resistance genes to carbapenems.

The whole-genome sequencing data revealed the occurrence of *bla*_*TEM*–1_ in the majority (*n* = 73; 92.4%) of the ampicillin-resistant isolates. The genes encoding extended-spectrum beta-lactamases (ESBLs) and AmpC-type cephalosporinases were identified in 18 (22.5%) *E. coli* belonging to 14 STs: *bla*_*SHV*–12_ was present in five isolates (ST58, ST69, ST359, and ST1011), *bla*_*CTX*–*M*–1_, *bla*_*TEM*–30_, and *bla*_*TEM*–135_ in two each (respectively, ST617, ST1611, ST154, ST617, ST93, and ST5979), single isolates carried *bla*_*CTX*–*M*–15_ (ST767), or *bla*_*TEM*–52*C*_ (ST117) and *bla*_*CMY*–2_ was present in six strains (ST48, ST58, ST155, ST398, and ST1011) (Figure 7). Fifteen isolates carried extended-spectrum cephalosporin (ESC) resistance gene in combination with bla_*TEM*–1_. Two isolates, U15_0035X (ST767) and U16_0016X (ST617), possessed simultaneously two ESC resistance genes, respectively, *bla*_*CTX*–*M*–15_ with *bla*_*CMY*–2_ and *bla*_*CTX*–*M*–1_ with *bla*_*TEM*–30_. The swine isolate (U15_0035X) was the only one carrying the *bla*_*CTX*–*M*–15_ gene.

Analysis of the genetic background of resistance to quinolones showed chromosomal mutations in the quinolone resistance-determining region (QRDR) of topoisomerase genes in 63.8% (*n* = 51) isolates, resulting in amino acid substitutions in the *gyrA* subunit [Ser83→Leu (*n* = 48); Asp87→Ans (*n* = 40), Asp87→Tyr (*n* = 2)], *parC* [Ser80→Ile (*n* = 37), Ser80→Arg (*n* = 4); Ser57→Thr (*n* = 1); Glu84→Gly (*n* = 3), Glu84→Lys (*n* = 2)], and *par*E [Leu416→Phe (*n* = 2), Leu460→Asp (*n* = 1)]. The *gyrB* gene remained unaltered. Several silent mutations irrelevant for quinolone resistance were also noted. Different patterns combining up to four simultaneous amino acid substitutions were noted among tested isolates with a combination of mutations in *gyrA* S83L, *gyrA* D87N, and *parC* S80I being the most frequent (*n* = 30) (Figure 7). Plasmid-mediated quinolone resistance (PMQR) genes were detected in 23 isolates, namely *qnrS1* (*n* = 12) and *qnrB19* (*n* = 11) (Figure 7). Four of the PMQR carriers also harbored QRDR chromosomal mutations. Eight isolates carried both ESBL/AmpC and PMQR determinants. The *aac*(*6′)Ib-cr* gene, conferring resistance to both quinolones and aminoglycosides, was identified in two strains, occurring along with *qnrS1* (U15_0035X), or the set of mutations in *gyrA* (S83L and D87N) and *parC* (S80I and E84G) (U14_0810). A 21.3% portion of the isolates carrying quinolone resistance mechanisms were also confirmed as ESBL- or AmpC-producers.

A variety of gentamicin resistance genes was identified. The sequences revealed genes coding N-acetyltransferases catalyzing acetyl CoA-dependent acetylation of an amino group, like *aac(3′)-IIa* (*n* = 8) and *aac(3′)-IId* (*n* = 5), and O-phosphotransferases (APH) catalyzing ATP-dependent phosphorylation of a hydroxyl group, namely *aph*(*3′)-Ia* (*n* = 14) (Supplementary Table S1). Overall, there was 93.8% genotype–phenotype correlation for gentamicin resistance. The presence of genes coding adenyltransferases [*aadA1, aadA2, aadA5, aadA24*, and *ant(2”)-Ia*] was identified in 50 isolates (Supplementary Table S1). WGS data showed the occurrence of three genes responsible for macrolide resistance: *mph*(B), *mph*(A), and *msr*(E)-*mph*(E) in single isolates with MICs equal to 8, 16, and 32 mg/L, respectively. Sixty isolates were resistant to sulfonamides due to *sul1* (*n* = 32), *sul2* (*n* = 39), or *sul3* (*n* = 18). In 5 isolates all three genes occurred simultaneously, while in 23 a set of two genes was found with *sul*1 and *sul2* being the most frequent (*n* = 19). Of the 49 trimethoprim-resistant *E. coli*, 45 harbored at least one of the following genes: *dfrA1* (*n* = 33), *dfrA12* (*n* = 2), *dfrA14* (*n* = 5), *dfrA15* (*n* = 1), *dfrA16* (*n* = 1), and *dfrA17* (*n* = 5).

At least one of the tetracycline resistance genes *tet*(A) or *tet*(B) was carried by 73 isolates, these genes being found, respectively in 60 and 18 *E. coli*. In five isolates both genes were detected and *tet*(M) was additionally identified in one of them. Overall, there was 100% genotype–phenotype correlation for tetracycline resistance. In 31 isolates the presence of *catA1* (*n* = 13), *catB3* (*n* = 2), *cmlA1* (*n* = 18), and *floR* (*n* = 10) was confirmed. In four isolates the resistance genes were present despite a lack of phenotypic resistance to chloramphenicol. Two of them possessing the *cmlA1* gene had MIC = 16 mg/L, one isolate had two point mutations in *cmlA1* and in the last case a fragment of the *catB3* gene was missing (short contig length).

### Plasmids and Location of the *mcr-1* Gene

*Escherichia coli* positive for *mcr-1* carried a wide variety of plasmid incompatibility group replicons in different proportions and ranging from 4 up to 11 replicons per strain (Supplementary Table S1). The most frequent were: IncFIB (AP001918) (*n* = 64), ColRNAI (*n* = 47), Col (MG828) (*n* = 43), IncFII (*n* = 40), IncI1 (*n* = 30), p0111 (*n* = 24), IncFIC (FII) (*n* = 21), Col156 (*n* = 17), IncX1 (*n* = 17), IncHI2A (*n* = 16), IncQ1 (*n* = 14), and IncN (*n* = 9). Plasmid replicons of all other identified plasmids are noted in Supplementary Table S1.

**FIGURE 8:**
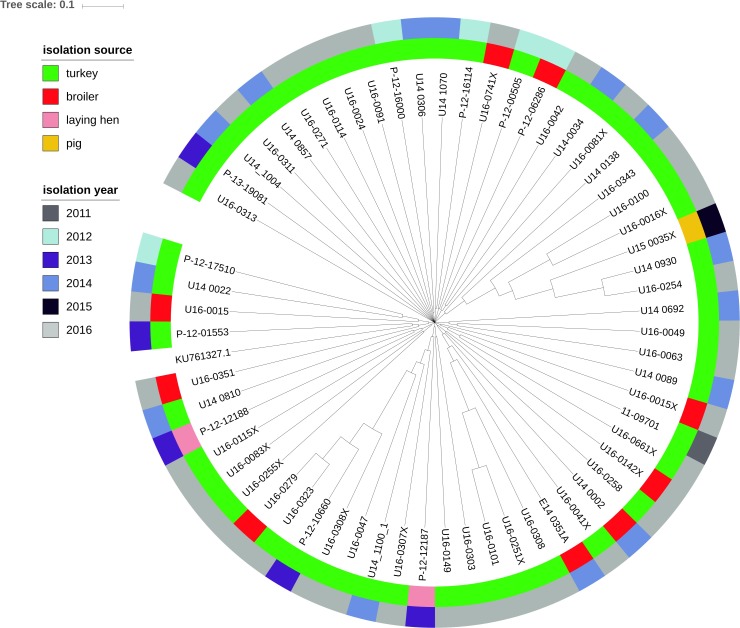
Phylogenetic relationship of contigs with *mcr-1* and IncX4 plasmid replicon extracted from the genomes of 61 *E. coli* isolates by source and year of *E. coli* isolation. Complete sequence of IncX4 plasmid with accession number KU761327.1 from GenBank was used as the reference.

Sixty-one isolates out of the 80 *mcr-1*–positive *E. coli* (76.3%) harbored plasmids of the IncX4 group with the replicon located on the same contig of the *mcr-1* gene (hereafter IncX4–*mcr-1* contigs). In most cases, the *mcr-1* gene was the only resistance gene found on IncX4–*mcr-1* contigs, which ranged in size from 10772 to 39252 bp. The isolates U16_0149 and U16_0323 also contained the *qnrS1* and *bla*_*TEM*–1_ genes and IncX1 replicon located on the IncX4–*mcr-1* contig (contig sizes 76785 bp and 69841 bp, respectively). IncX4–*mcr-1* contigs were of high sequence similarity and clustered independently of the sample isolation source and sampling year (Figure 8).

In five of the *mcr-1*–positive *E. coli* isolates (6.3%), IncHI2 plasmids were found to be *mcr-1* carriers. In 4 out of 5 cases the *mcr*-carrying plasmid was identified using PacBio data as it was not possible to link the plasmid replicon with the *mcr-1* gene using only short Illumina reads. All the IncHI2–*mcr-1* plasmids were subtyped as pST4. On the IncHI2–*mcr-1* contig (234213 bp) of the U16_0565X isolate additional resistance genes were identified as follows: *aadA1*, *aadA2*, *bla*_*TEM*–1B_, *catA1*, *cmlA1*, *sul1*, and *tet*(A). This isolate possessed the *bla*_*TEM*–52*C*_ gene located on the other contig (8917 bp). On the IncHI2–*mcr-1* contig (235356 bp) of U16_0288X, *aac(3)-IIa, aadA1, aadA2*, bla_*TEM*–1C_, *sul1, cmlA1, dfrA1, tet*(A), *aph(3″)-Ib*, and *aph(6)-Id* were also found. The presence of the *aph(3′*)-*Ia* gene was confirmed on the relevant contig (201917 bp) of U16_0579. No other plasmid replicons except IncHI2 were annotated on those contigs.

Three isolates (3.7%) possessed both the IncHI2 and IncX4 replicons, but *mcr-1* was associated with IncX4. In one strain (U16_0259) a chromosomal location of the *mcr-1* gene was confirmed (data not shown). In 15.0% (*n* = 12) of isolates no plasmid replicons were found on contigs carrying the *mcr-1* gene (ranging in size from 2587 to 57048 bp) but the presence of the IncX4 or IncHI2 replicon in the assembly was confirmed. A curiosity is that in two *E. coli* (ID U16_0115 and U16_0115X) isolated from the same sample, the *mcr-1* genes were located on different incompatibility group plasmids (IncX4 or IncHI2).

### Virulence Genes

The virulence genes were variable among isolates (Supplementary Table S1). Of the 80 *E. coli* sequences, six contained one virulence gene, whereas the remainder carried up to 10 virulence genes. The most common were: *gad* (*n* = 72), *iss* (*n* = 62), *iroN* (*n* = 56), *lpf*A (*n* = 41), *cma* (*n* = 28), *mchF* (*n* = 25), *astA* (*n* = 19), *air* (*n* = 15), *eilA* (*n* = 13), and *tsh* (*n* = 10), whereas *cba, celb, ireA, vat, cap*U*, iha*, and *mcmA* were found in single isolates. We found no correlation of virulence genes with sample source or sampling year. Isolates were characterized with different sets of virulence genes. Notably, one isolate (U14_0002) presented a unique set of virulence genes (*cif*, *eae, espA, espB, espF, nleB, tccP*, and *tir*) that designated it as atypical enteropathogenic *E. coli* (aEPEC) group (Supplementary Table S1).

## Discussion

Based on screening of colistin MIC values in *E. coli* derived from various monitoring programs on AMR in 2011–2016, we collected extensive information about the occurrence of the *mcr-1* gene in *E. coli* isolated from food-producing animals in Poland. Detailed characterization of *mcr-1*–positive isolates from several hosts, different geographical locations, and a range of sampling years included analysis of the phenotypic AMR to a broad range of antimicrobials and its genetic background, the presence of virulence genes, plasmid replicons, and ST identification.

Many European countries reported the occurrence of colistin resistance in *E. coli* deriving from both humans and animals (Hasman et al., 2015; Irrgang et al., 2016; Malhotra-Kumar et al., 2016; Perrin-Guyomard et al., 2016; Carattoli et al., 2017; Duggett et al., 2017; Hartl et al., 2017; Kawanishi et al., 2017; Apostolakos and Piccirillo, 2018). In Poland, we observed a slight increase in colistin resistance in *E. coli* and also in the prevalence of *mcr*-positive isolates originating from healthy livestock from 0.7 to 1.7% and 0.2 to 3.7%, respectively in the analyzed time frame, irrespective of the animal of origin. The overall occurrence of colistin resistance in turkeys was higher than in chickens but it still remained low compared to data from some European countries (European Food Safety Authority [EFSA], 2018).

*Escherichia coli* totaling 53 *mcr*-positive *E. coli* were identified after including isolates with MIC_*colistin*_ = 2 mg/L, which is the EUCAST epidemiological cut-off delimiting the wild-type population. Applying this criterion, the prevalence was 3.7% *mcr-1*–positive *E. coli* rather than 0.8% in Poland in 2016. Detection of the *mcr* gene in wild-type isolates was reported (Fernandes et al., 2016; Lentz et al., 2016; Hadjadj et al., 2017; Wang et al., 2017; Zhou et al., 2017) and might result from a non-functional *mcr-1* gene (Terveer et al., 2017). Some reports indicate the possibility of deactivation of *mcr-1* by insertion of an IS1294b element and its reactivation by the loss of that element under colistin selection pressure (Zhou et al., 2018). In the current study, all of the *mcr-1* had the typical sequence of the *mcr-1*.*1* gene. In some cases, the wild-type concentration MIC_*colistin*_ = 2 mg/L could result from a limitation in the MIC determination method where one dilution step difference is permissible. It should therefore be considered during selection of suspected isolates. Except for one isolate, the presence of *mcr-1* was not associated with a high level of resistance (MIC > 4 mg/L) to colistin and the presence of a chromosomal resistance mechanism in one of the isolates did not lead to elevated colistin MIC values either (MIC = 2 mg/L). There was a noted presence in Brazil of the *mcr-1* gene in wild type isolates derived from poultry confirmed as never exposed to polymyxin during their entire lives (Lentz et al., 2016).

In some cases the lack of genotype–phenotype correlation in isolates with resistance genes but without phenotypic resistance to chloramphenicol could result from the limitation of the MIC method. In others the reason could be substitutions found in the relevant gene. In an isolate carrying the *catB3* gene the lack of genotype–phenotype correlation could not be identified due to lack of a fragment gene at one end of the contig.

Despite several *mcr*-types and their variants being described in isolates from animals across Europe (Rebelo et al., 2018), our study suggests only *mcr-1*.*1* being present in Polish livestock, the first cases dating back to 2011. For yet unknown reasons, but in concordance with data from Germany and France, the highest occurrence of *mcr-1*–positive *E. coli* was detected in turkeys (Irrgang et al., 2016; Perrin-Guyomard et al., 2016). We speculate it could be related to the longer life span of these animals compared to chickens, and consequently to a longer length of exposure to selective pressure favoring antibiotic resistance. Colistin is used for treatment of gastrointestinal infections in animals, but in some countries low doses may be used as a growth promoter (Kempf et al., 2013; Fernandes et al., 2016). However, this practice is not allowed in Poland or the other EU countries (European Medicine Agency [EMA] and European Surveillance of Veterinary Antimicrobial Consumption [ESVAC], 2017). The proliferation of *mcr-1*–carrying *E. coli*, only occasionally found in 2011 but reaching a case count of several dozen by 2016, raises the question of the effects of excessive colistin use in animal husbandry. Worth noting is that in Poland, unlike other animal species, most of the turkey population is raised from imported one-day-old poults or hatching eggs and it might be an additional way for resistant isolates to be introduced to Polish farm environments. Some research indicates that the introduction of resistant bacteria may have been through imported breeding animals (Mo et al., 2014).

Horizontal transfer via plasmids plays an important role in the dissemination of antibiotic resistance genes. The IncX4 plasmid is considered one of the most prevalent carriers of the *mcr-1* gene in *Enterobacteriaceae* (Johnson et al., 2012; Matamoros et al., 2017; Sun et al., 2017). Our study showed that *mcr-1* was associated with IncX4 plasmids in the vast majority (76.3%) of isolates, and with IncHI2 plasmids, another well-known *mcr-1* vector (Matamoros et al., 2017; Sun et al., 2018) in a few (6.3%) isolates. The fact that in one sample two different *E. coli* were found with *mcr-1* located on different plasmids (IncX4 and IncHI2) might evidence a parallel route of resistance spread but we cannot exclude transfer across the plasmids. Furthermore, occurrence of the *mcr-1* gene on the chromosome shows that plasmid-mediated colistin resistance genes might become fixed into specific *E. coli* populations and spread vertically.

Most of the tested isolates were genetically unrelated, which has also been observed in other reports on *mcr-1*–positive *E. coli* (Veldman et al., 2016). One of the reported *E. coli* ST 10, identified in 3 isolates, has been previously described in relation to *mcr-1* (Yang et al., 2017), and is considered a reservoir of this gene (Matamoros et al., 2017). The STs exhibited genetic diversity and were not related to animal source, geographic area, or isolation year. The identification of the same ST (i.e., ST919, ST354, and ST1564) in strains deriving from the same animal source but isolated in different years, or even in strains isolated from different species and in some cases harboring the *mcr-1* gene on different plasmids proves the wide dissemination of plasmid-mediated colistin resistance over the whole country. The study shows, in the light of the ESVAC data on colistin sales (European Medicine Agency [EMA] and European Surveillance of Veterinary Antimicrobial Consumption [ESVAC], 2017), that the phenomenon is probably a result of wide colistin selection pressure and plasmid dissemination, and not due to the spread of specific bacterial clones (El Garch et al., 2017; Wang et al., 2017). In Poland, sales of colistin still remain above the maximum sale target (European Medicine Agency [EMA] and European Surveillance of Veterinary Antimicrobial Consumption [ESVAC], 2018). External introduction, transmission of plasmids, and dissemination under selection pressure create the potential for the *mcr-1* gene to become established in Polish food-producing animals.

Of significance is that almost all *mcr-1*.*1–*positive isolates were MDR including the compounds considered CIA (World Human Organization [WHO], 2017). They carried a range of genes encoding resistance to cephalosporins and quinolones. Some reports have demonstrated the presence of the *mcr-1* gene together with ESBL genes (Robin et al., 2017; Yamaguchi et al., 2018). Therefore *mcr-1*–positive *E. coli* should be considered a reservoir not only of the colistin resistance gene, but also of those of PMQR, and ESBL or sets of other resistance genes carried along with *mcr-1* on some plasmids. This is supported by our finding of the genes encoding for resistance to beta-lactams, including cephalosporins, aminoglycosides, or sulphonamides located on the same contig as *mcr-1*.*1* and IncHI2 replicon. This is a serious concern for veterinary medicine and also for human health since direct transmission of resistant isolates from animals to humans has been confirmed (Marshall and Levy, 2011). The genes found in the current study did not differ from the ones identified previously in *E. coli* occurring in the healthy animal population (Wasyl, 2014; Lalak et al., 2016). The *bla*_*CTX*–*M*–15_ gene, which occurs in isolates responsible for nosocomial infections in Poland (Empel et al., 2008) was found in this study in only a single pig isolate.

The aEPEC (atypical enteropathogenic *Escherichia coli*) isolates are a cause of diarrhea in both humans and animals (Afset et al., 2004; Almeida et al., 2012). Here, in the collection of non-clinical *E. coli* isolates from healthy animals, we identified *mcr-1*.*1* in a single chicken strain surprisingly carrying several virulence determinants of the aEPEC phenotype, namely EAST1, cell cycle inhibiting factor, intimin adherence protein Eae, secreted proteins EspA, EspB, and EspF type III secretion system effector NleB, Tir-cytoskeleton coupling protein, and translocated intimin receptor Tir. The strain carried also additional AMR genes combining to afford resistance to 4 classes. Since the *mcr-1*.*1*–positive, multidrug resistant aEPEC should be considered a vector of both resistance determinants and pathogens, this finding is worrisome for successive treatment of animals or humans.

## Conclusion

The results highlight that poultry, especially turkeys, can be an important reservoir of *mcr-1*.*1*–carrying *E. coli* strains in Poland. Our findings indicate an increasing occurrence of *mcr-1.1* in *E. coli* from turkeys and, to a lesser extent, chickens in Poland from 2011 to 2016, whereas cases in pigs and cattle appear to be sporadic in the study period. The *mcr-1*.*1* gene occurred mainly on the IncX4 and IncHI2 plasmids in a wide diversity of *E. coli* harboring multiple resistance genes, virulence genes, and various plasmid replicons. Thus, dissemination of *mcr-*positive plasmids is a probable pathway for plasmid-mediated colistin resistance to spread in food-producing animals. The impressive genetic diversity of isolates as well as the association of colistin resistance with particularly relevant phenotypes (e.g., third-generation cephalosporin and fluoroquinolone resistance as well as aEPEC) call for urgent reduction in the use of colistin to avoid further selection of co-resistance in *E. coli* in animal production and possible animal and public health consequences. Definitely excluding isolates that are currently considered wild-type might contribute to silent dissemination of the *mcr*-positive ones. Great attention should be given to continuous phenotypic and genotypic surveillance of AMR and data collection in both human and veterinary settings, thus enabling intervention to counteract any rapid dissemination of *mcr-1*.*1*–positive *E. coli*.

## Author Contributions

MZ and DW designed the experiments. MZ, PS, DW, and AZ-B analyzed the resistance and genotypic data. MZ, PS, and AZ-B prepared the tables and figures. MZ, PS, and DW prepared the manuscript. All authors discussed the results, reviewed and edited the manuscript, read, and approved the final version of the manuscript.

## Disclaimer

The conclusions, findings and opinions expressed in this scientific paper reflect only the view of the authors and not the official position of the European Food Safety Authority.

## Conflict of Interest Statement

The authors declare that the research was conducted in the absence of any commercial or financial relationships that could be construed as a potential conflict of interest.

## Footnotes

1 http://bip.urpl.gov.pl/pl/biuletyny-i-wykazy/produkty-lecznicze-weterynaryjne

2 https://cge.cbs.dtu.dk/services/
