# Corrigendum: Occurrence and Characterization of *mcr-1*-Positive *Escherichia coli* Isolated From Food-Producing Animals in Poland, 2011–2016

**DOI:** 10.3389/fmicb.2019.02816

**Published:** 2019-12-04

**Authors:** Magdalena Zając, Paweł Sztromwasser, Valeria Bortolaia, Pimlapas Leekitcharoenphon, Lina M. Cavaco, Anna Ziȩtek-Barszcz, Rene S. Hendriksen, Dariusz Wasyl

**Affiliations:** ^1^Department of Microbiology, National Veterinary Research Institute, Puławy, Poland; ^2^Department of Omics Analyses, National Veterinary Research Institute, Puławy, Poland; ^3^Research Group for Genomic Epidemiology, European Union Reference Laboratory for Antimicrobial Resistance, WHO Collaborating Centre for Antimicrobial Resistance in Foodborne Pathogens and Genomics, National Food Institute, Technical University of Denmark, Lyngby, Denmark; ^4^Statens Serum Institute, Copenhagen University, Copenhagen, Denmark; ^5^Department of Epidemiology, National Veterinary Research Institute, Puławy, Poland

**Keywords:** WGS, *mcr-1*, colistin resistance, aEPEC, food animal, IncX4, IncHI2

In the original article the disclaimer was not included in the published article. The disclaimer appears below.

## Disclaimer

The conclusions, findings and opinions expressed in this scientific paper reflect only the view of the authors and not the official position of the European Food Safety Authority.

The authors apologize for this error and state that this does not change the scientific conclusions of the article in any way. The original article has been updated.

