# Supplementary material for: Occurrence and Characterization of mcr-1-Positive Escherichia coli Isolated From Food-Producing Animals in Poland, 2011–2016
Source: Front Microbiol. 2019 Aug 8;10:1753. doi: 10.3389/fmicb.2019.01753 (PMC6694793; doi:10.3389/fmicb.2019.01753)
Supplement: Supplementary file 1 [file Table_1.DOCX]

Supplementary Material

# Supplementary Table S1. Detailed information about tested isolates. „–like” means not 100% identity with the reference or not full length of the sequence. Bolded plasmid name means replicon found on the same contig as the *mcr-1.1* gene. Isolates marked with “*”, “**”, “#”, “##” mean deriving from the same sample.

| **Strain ID** | **Year** | **Source** | **MLST type** | **Colistin MIC (mg/L)** | **Plasmids replicons** | **Virulence genes** | **Resistance genes** | **ENA number** |
| --- | --- | --- | --- | --- | --- | --- | --- | --- |
| **11-09701** | 2011 | turkey | ST-533 | 4 | *Inc*FII, *Inc*FIB(AP001918), ***Inc*X4**, *Col*(MG828) | *cma, gad, iss, lpfA* | *bla*_TEM-1B_, *mcr-1.1, mdf*(A)-like | ERS2055657 |
| **11-14155** | 2011 | turkey | ST-58 | >4 | *Inc*FIC(FII), *Inc*FIA, *Inc*HI2, *Inc*FIB(AP001918), *Inc*HI2A, *Col*(MG828), *Inc*Q1, TrfA, *Inc*B/O/K/Z | *cma, gad, iroN, iss, lpfA, mchF, tsh* | *aac(3)-IIa*, *aadA1*, *aadA2*, *aph(3’)-Ia*-like, *aph(3’’)-Ib*, *aph(6)-Id*, *bla*_TEM-1A_, *catA1*-like, *cmltA1*-like, *dfrA1*-like, *mcr-1.1*, *mdf*(A)-like, *sul1*, *sul2*, *sul3*, *tet*(A) | ERS2055658 |
| **P-12-00505** | 2012 | turkey | ST-919 | 4 | *Inc*FII, *Inc*FIB(AP001918, *Inc*N, ***Inc*X4**, *Col*(MG828) | *gad, iha, iroN, iss, vat* | *aph(6)-Id, aph(3’’)-Ib*-like, *bla*_TEM-1B_-like, *mcr-1.1*, *mdf*(A)-like, *qnrS1*, *tet*(A) | ERS2055659 |
| **P-12-00759** | 2012 | turkey | ST-93 | 4 | *Inc*FIC(FII), *Inc*HI2, *Inc*FIB(AP001918), *Inc*HI2A, *Inc*X1, TrfA, *Inc*B/O/K/Z | *capU, gad, ireA, iroN, iss, mchF, tsh* | *aadA1*-like, *aadA2*, *aph(3’’)-Ib*, *aph(6)-Id*-like, *bla*_TEM-135_, *catA1*-like, *cmltA1*-like, *dfrA1*, *floR*-like, *mcr-1.1*, *mdf*(A)-like, *qnrS1*, *sul1*, *sul2*, *sul3*, *tet*(A)-like | ERS2055654 |
| **P-12-06286** | 2012 | broiler | ST-57 | 4 | *Inc*HI2, *Inc*FIC(FII), *Inc*HI2A, *Inc*I1, *Inc*FIB(AP001918), *Inc*FIA, *Inc*N, *Inc*X1, TrfA, *Col*(MG828), ***Inc*X4**, *Col*RNAI | *gad, iss, lpfA* | *bla*_TEM-1B_, *catA1*-like, *mcr-1.1*, *mdf*(A)-like, *tet*(A) | ERS2055665 |
| **P-12-12952** | 2012 | turkey | ST-1196 | 4 | *Inc*FII, *Inc*FIB(AP001918), ***Inc*X4**, *Col*(MG828) | *cma, ireA, iroN, iss, mchF* | *bla*_TEM-1B_, *mcr-1.1*, *mdf*(A)-like, *tet*(A) | ERS2055653 |
| **P-12-16000** | 2012 | turkey | ST-58 | 4 | *Inc*Q1, ***Inc*X4**, *Col*RNAI | *gad* | *aac*(3)-IId-like, *bla*_CMY-2_, *bla*_TEM-1B_, *mcr-1.1*, *mdf*(A)-like, *sul2*-like, *tet*(B) | ERS2055660 |
| **P-12-16114** | 2012 | turkey | ST-949 | 4 | *Inc*FII, *Inc*I1, *Inc*FIB(AP001918), *Inc*I2, *Col*RNAI, ***Inc*X4**, *Col*(MG828) | *cma, gad, lpfA* | *aph(3’’)-Ib*-like, *aph(6)-Id*, *bla*_TEM-1C_-like, *mcr-1.1*, *mdf*(A)-like, *tet*(A)-like | ERS2055661 |
| **P-12-17510** | 2012 | turkey | ST-624 | 4 | *Inc*I1, *Col*(MG828), *Col*RNAI, *Col*156, *Inc*X1, p0111, ***Inc*X4** | *gad, lpfA* | *aadA1*, *aph(3’)-Ia*-like, *aph(3’’)-Ib*, *aph(6)-Id*,*bla*_TEM-1C_, *dfrA1*, *mcr-1.1*, *mdf*(A)-like, *sul2*-like, *tet*(B)-like | ERS2055664 |
| **P-12-01553** | 2013 | turkey | ST-154 | 4 | *Inc*HI2A, *Inc*FII, *Inc*HI2, TrfA, p0111, *Col*RNAI | *gad, lpfA* | *aadA1*, *aadA2*, *bla*_TEM-30_, *cmltA1*-like, *dfrA1*, *mcr-1.1*, *mdf*(A)-like, *sul2*, *sul3*, *tet*(A) | ERS2055663 |
| **P-12-10660** | 2013 | turkey | ST-7315 | 4 | *Inc*I1, *Inc*FIB(AP001918), *Inc*FII(pCoo), *Col*8282, *Col*RNAI, *Col*156, ***Inc*X4**, *Col*(MG828) | *celb, cma, gad, iroN, iss, lpfA* | *aac*(3)-*IId*-like, *aadA2*-like, *aph(3’)-Ia*-like, *aph(3’’)-Ib*, *aph(6)-Id*, *bla*_TEM-1B_, *catA1*-like, *dfrA1*2, *mcr-1.1*, *mdf*(A)-like, *sul1*, *sul2*, *tet*(A)-like | ERS2055662 |
| **P-12-12187** | 2013 | laying hen | ST-359 | 4 | *Inc*FII, *Inc*I1, *Inc*FIB(AP001918), *Inc*N, *Col*156, ***Inc*X4** | *gad, iroN, iss, lpfA, mchF* | *aadA1*, *aph(3’)-Ia*-like, *aph(3’’)-Ib*, *aph(6)-Id*, *bla*_TEM-1B_, *dfrA1*, *floR*-like, *mcr-1.1*, *mdf*(A)-like, *sul1*-like, *sul2*, *tet*(B) | ERS2055650 |
| **P-12-12188** | 2013 | laying hen | ST-359 | 4 | *Inc*FII, *Inc*FIB(AP001918), *Col*(MG828), ***Inc*X4**, *Col*RNAI | *air, eilA, gad, ireA, iroN, iss, lpfA, mchF, mcmA* | *aadA1*, *bla*_TEM-1B_-like, *dfrA1*, *floR*-like, *mcr-1.1*, *mdf*(A)-like, *sul1*-like, *sul2* | ERS2055656 |
| **P-13-19081** | 2013 | turkey | ST-617 | 4 | *Inc*FIC(FII), *Inc*FIB(AP001918), *Inc*B/O/K/Z, *Col*RNAI, *Col*156, ***Inc*X4**, *Col*(MG828) | *capU, gad, iroN, iss, mchF* | *aph(3’’)-Ib*, *aph(6)-Id*-like, *bla*_TEM-1C_, *floR*-like, *mcr-1.1*, *mdf*(A)-like, *sul1*, *sul2*, *tet*(B)-like | ERS2055651 |
| **E14_0154B** | 2014 | *cat*tle | ST-88 | 2 | *Inc*FIC(FII), *Inc*FIB(AP001918), *Inc*Y, *Inc*X4, *Col*RNAI, *Inc*F[F18:A-:B1] | *gad, iroN, iss, lpfA, mchF, mcmA* | *aadA2*4-like, ant(2'')-Ia, *bla*_OXA-1_, *catA1*-like, *floR*-like, *mcr-1.1*, *mdf*(A)-like, *sul1*, *sul2*, *tet*(B) | ERS2055592 |
| **E14_0351A** | 2014 | broiler | ST-398 | 2 | *Inc*FII, *Inc*I1, *Inc*FIB(AP001918), p0111, ***Inc*X4**, *Col*RNAI, IncF[F24:A-:B6], IncI1[ST-36] | *cma, iroN, iss* | *aadA1*, *bla*_TEM-1B_, *dfrA1*, *mcr-1.1*, *mdf*(A)-like, *qnrB19*-like, *sul1*, *tet*(A) | ERS2055587 |
| **U14_0002** | 2014 | broiler | ST-189 | 2 | *Inc*FII(29), *Inc*Y, *Col*RNAI, ***Inc*X4**, *Col*(MG828) | *astA, cif, eae, espA, espB, espF, gad, nleB, tccP, tir* | *aph(3’)-Ia*-like, *aph(3’’)-Ib, aph(6)-Id, bla*_TEM-1B_, *mcr-1.1*, *mdf*(A)-like, *sul1*, *sul2*, *tet*(A), *tet*(B) | ERS2055586 |
| **U14_0022** | 2014 | turkey | ST-1564 | 2 | *Inc*FIC(FII), *Inc*I1, *Inc*FIB(AP001918), *Inc*X1, p0111, ***Inc*X4**, *Col*RNAI | *gad, iroN, iss, mchF, tsh* | *aadA1*-like, *bla*_TEM-1B_, *mcr-1.1*, *mdf*(A)-like, *qnrS1*, *sul2*, *tet*(A) | ERS2055591 |
| **U14_0076** | 2014 | turkey | ST-753 | 2 | *Inc*HI1B(CIT), p0111, *Inc*X4, *Col*RNAI | *air, eilA, gad* | *aadA1*, *aadA2*, *aph(3’)-Ia*-like, *bla*_TEM-1B_-like, *cmltA1*-like, *mcr-1.1*, *mdf*(A)-like, *qnrB19*, *sul3*, *tet*(A) | ERS2055597 |
| **U14_0089** | 2014 | turkey | ST-155 | 2 | *Inc*FIC(FII), *Inc*I1, *Inc*FIB(AP001918), ***Inc*X4** | *gad, iroN, iss, lpfA, mchF* | *aph(3’’)-Ib*-like, *aph(6)-Id*-like, *bla*_TEM-1B_, *dfrA14*-like, *mcr-1.1*, *mdf*(A)-like, *sul2*, *tet*(A) | ERS2055581 |
| **U14_0138** | 2014 | turkey | ST-624 | 2 | *Inc*FIB(pLF82), *Inc*FII, *Inc*FIB(AP001918), p0111, ***Inc*X4** | *air, astA, eilA, gad, ireA, iroN, iss, lpfA, mchF* | *aadA1*, *aph(3’)-Ia*-like, *aph(3’’)-Ib*, *aph(6)-Id*, *sul2*-like, *bla*_TEM-1C_, *dfrA1*, *mdf*(A)-like, *mcr-1.1*, *mdf*(A)-like, *tet*(B) | ERS2055594 |
| **U14_0306** | 2014 | turkey | ST-155 | 2 | *Inc*FIC(FII), *Inc*I1, *Inc*FIB(AP001918), ***Inc*X4** | *gad, iroN, iss, lpfA, mchF* | *aph(3’’)-Ib*-like, *aph(6)-Id*-like, *bla*_TEM-1B_, *dfrA14*-like, *mcr-1.1*, *mdf*(A)-like, *sul2*, *tet*(A) | ERS2055588 |
| **U14_0418** | 2014 | turkey | ST-354 | 2 | *Inc*HI2A, *Inc*FII, *Inc*HI2, *Inc*FIB(AP001918), *Inc*FII(29), TrfA, p0111, *Col*(MG828), *Inc*Q1, *Col*RNAI | *air, astA, cba, cma, eilA, gad, iroN, iss, lpfA, mchF* | *aac(3)-IIa*, *aadA1*, *aadA2*, *aph(3’)-Ia*-like, *aph(3’’)-Ib*, *aph(6)-Id*, *bla*_TEM-1C_, *catA1*-like, *cmltA1*-like, *dfrA1*, *mdf*(A)-like, *mcr-1.1*, *sul1*, *sul2*, *sul3*, *tet*(A) | ERS2055598 |
| **U14_0692** | 2014 | turkey | ST-10 | 2 | *Inc*FII(29), *Col*(MGD2), ***Inc*X4**, *Col*RNAI | *gad* | *aac(3)-IIa*, *aadA1*, *aph(3’’)-Ib*, *aph(6)-Id*, *bla*_TEM-1D_-like, *dfrA1*, *mcr-1.1*, *mdf*(A)-like, *tet*(A) | ERS2055596 |
| **U14_0810** | 2014 | turkey | ST-156 | 2 | *Inc*FIB(AP001918), *Inc*FIC(FII), *Col*156, ***Inc*X4** | *astA, cba, cma, gad, iroN, iss, lpfA* | *aac*(6')*Ib-cr-*like, *aac(6')-*Ib*3-like bla*_TEM-1B_, *catA1*-like, *cat*B3, *dfrA1*, *mcr-1.1*, *mdf*(A)-like, *sul1*, *tet*(B) | ERS2055589 |
| **U14_0857** | 2014 | turkey | ST-10 | 2 | *Inc*FIB(AP001918), *Inc*FII(pCoo), *Inc*Y, *Col*156, ***Inc*X4** | *astA, gad, iroN, iss* | *aph(3’’)-Ib*, *aph(6)-Id*, *bla*_TEM-1B_, *dfrA14*, *mcr-1.1*-like, *mdf*(A)-like, *sul2*, *tet*(A) | ERS2055593 |
| **U14_0930** | 2014 | turkey | ST-93 | 2 | *Inc*FIC(FII), *Inc*FIB(AP001918), ***Inc*X4**, *Col*RNAI | *capU, cma, gad, iha, iroN, iss* | *bla*_TEM-1B_-like, *mcr-1.1*, *mdf*(A)-like, *tet*(A) | ERS2055584 |
| **U14_1004** | 2014 | turkey | ST-617 | 2 | *Inc*FIB(AP001918), *Inc*FII(pCoo), *Col*RNAI, ***Inc*X4**, *Col*(MG828) | *astA, gad, iroN, iss* | *aph(3’’)-Ib*-like, *aph(6)-Id*, *bla*_TEM-1B_, *mcr-1.1*, *sul2*, *tet*(A) | ERS2055583 |
| **U14_1070** | 2014 | turkey | ST-617 | 2 | *Inc*FIB(AP001918), *Inc*FII(pCoo), *Col*RNAI, ***Inc*X4**, *Col*(MG828) | *astA, gad, iroN, iss* | *aph(3’’)-Ib*-like*, bla*_TEM-1B_, *mcr-1.1*, *mdf*(A)-like*, sul2*, *tet*(A) | ERS2055590 |
| ***U14_1100_1** | 2014 | turkey | ST-2001 | 2 | *Inc*FII, *Inc*FIB(AP001918), *Inc*Y, *Col*RNAI, ***Inc*X4**, *Col*(MG828) | *air, cma, eilA, iroN, iss, lpfA* | *aph(3’’)-Ib*, *aph(6)-Id*, *bla*_TEM-1B_, *mcr-1.1*, *mdf*(A)-like, *tet*(B) | ERS2055432 |
| ***U14_1100_3** |  |  | ST-4598 | 2 | *Col*(MGD2), p0111, *Col*RNAI, *Inc*X4, *Col*(MG828) | *gad* | *bla*_TEM-1B_, *mcr-1.1*-like, *mdf*(A)-like, *tet*(A)-like | ERS2055433 |
| **U14_1192** | 2014 | turkey | ST-1126 | 2 | *Inc*HI2A, *Inc*FII, *Inc*HI2, *Inc*FIB(AP001918), TrfA, *Inc*Q1, *Col*RNAI, *Col*(MG828) | *cma, gad, iroN, iss, lpfA, mchF* | *aac(3)-IIa*, *aadA1*, *aph(3’’)-Ib*, *aph(6)-Id*, *bla*_TEM-1A_, *dfrA1*, *mcr-1.1*, *mdf*(A)-like, *sul1*, *sul2*, *tet*(A) | ERS2055585 |
| **U14-0034** | 2014 | turkey | ST-1851 | 4 | *Inc*FII, *Inc*FIB(AP001918), *Inc*N, *Inc*Y, *Col*(MG828), *Col*RNAI, ***Inc*X4**, *Inc*B/O/K/Z | *gad, iroN, iss, vat* | *aph(3’’)-Ib*-like, *aph(6)-Id*-like, *bla*_TEM-1B_-like, *floR*-like, *mcr-1.1*, *mdf*(A)-like, *qnrS1*, *sul2*, *tet*(A) | ERS2055652 |
| **U14-0397** | 2014 | turkey | ST-5979 | 4 | *Inc*HI1B(CIT), ***Inc*HI2**, *Inc*N, *Inc*HI2A, TrfA, p0111, *Inc*Q1, *Col*(MG828) | *air, eilA, gad* | *aac(3)-IIa*, *aadA1*, *aadA2*, *aph(3’’)-Ib*-like, *aph(6)-Id*, *bla*_TEM-135_-like, *cmltA1*-like, *dfrA1*, *mcr-1.1*, *mdf*(A)-like, *qnrS1*, *sul1*, *sul2*, *sul3*, *tet*(A) | ERS2055655 |
| **U14-0628** | 2014 | turkey | ST-410 | 4 | *Inc*FII, *Inc*I1, *Inc*FIB(AP001918), *Inc*N, *Col*(MG828), *Col*RNAI, *Col*156, *Col*E10, *Inc*X4, *Col*8282, *Inc*Q1 | *gad, ireA, iss, lpfA* | *aadA1*, *aph(3’’)-Ib*, *aph(6)-Id, bla*_TEM-1B_, *dfrA1*, *mcr-1.1*, *mdf*(A)-like, *sul1*, *sul2*, *tet*(A) | ERS2055666 |
| **U15_0035X** | 2015 | pig | ST-767 | 2 | *Inc*I1, *Inc*FII(pHN7A8), *Inc*FII, *Inc*FIB(AP001918), *Inc*FII(pRSB107), *Inc*FIA, *Inc*X1, *Col*RNAI, ***Inc*X4**, *Col*(MG828) | *astA, cba, cma, gad, iss, lpfA, tsh* | *aac*(6')-*Ib-cr*, *aadA1*, *aadA5*, *aph(3’)-Ia*-like, *bla*_CTX-M-15_, *bla*_OXA-1_, *bla*_TEM-1B_, *cat*B3-like, *dfrA1*, *dfrA17*, *mcr-1.1*, *mdf*(A)-like, *qnrS1*, *sul1*, *tet*(A), *tet*(B) | ERS2055595 |
| **U16_0579** | 2016 | broiler | ST-141 | 2 | *Inc*FIB(pLF82), *Inc*FIC(FII), ***Inc*HI2**, *Inc*FIB(AP001918), *Inc*HI2A, TrfA | *gad, iroN, iss, mchF, tsh* | *aadA2*4-like, *aph(3’)-Ia*-like, *dfrA1*-like, *mcr-1., mdf*(A)-like*1* | ERS2055582 |
| **** U16-0015** | 2016 | broiler | ST-48 | 2 | *Inc*FII, p0111, ***Inc*X4**, *Col*RNAI | *gad* | *aadA1*, *aadA2*, *bla*_CARB-2_, *bla*_TEM-1B_, *cmltA1*-like, *dfrA1*6, *mcr-1.1*, *mdf*(A)-like, *qnrB19*, *sul3*, *tet*(A)-like, *tet*(B) | ERS2055675 |
| **** U16-0015X** |  |  | ST-1011 | 2 | *Inc*FII, *Inc*I1, *Inc*FIB(AP001918), *Inc*Y, *Inc*Q1, ***Inc*X4** | *air, eilA, iroN, iss* | *aadA1*, *aph(3’’)-Ib*, *aph(6)-Id*, *bla*_SHV-12_, *bla*_TEM-1B_, *dfrA1*, *mcr-1.1*, *sul1*, *mdf*(A)-like, *sul2*, *tet*(A) | ERS2055643 |
| **U16-0016X** | 2016 | turkey | ST-617 | 2 | *Inc*FII, *Inc*I1, *Inc*FIB(AP001918), *Inc*X1, *Col*156, ***Inc*X4**, *Col*RNAI | *astA, gad, iroN, iss, mchF* | *aad*A5, *bla*_CTX-M-1_, *bla*_TEM-30_, *dfrA17*, *mcr-1.1*, *mdf*(A)-like, *tet*(B) | ERS2055676 |
| **U16-0024** | 2016 | turkey | ST-162 | 2 | *Inc*FII, *Inc*FIB(AP001918), p0111, *Col*(MG828), ***Inc*X4**, *Col*RNAI | *astA, cma, gad, iroN, iss, lpfA* | *aadA1*, *bla*_TEM-1B_-like, *cmltA1*-like, *dfrA1*5, *mcr-1.1*, *mdf*(A)-like, *qnrB19*-like, *sul3*, *tet*(A)-like | ERS2055673 |
| **U16-0041X** | 2016 | turkey | ST-1011 | 2 | *Inc*FII, *Inc*I1, *Inc*FIB(AP001918), *Inc*Q1, *Col*RNAI, *Col*156, ***Inc*X4**, *Col*(MG828) | *air, cma, eilA, iroN, iss* | *aac*(3)-IId-like, *aadA1*, *aph(3’’)-Ib*, *aph(6)-Id*, *bla*_CMY-2_, *bla*_TEM-1B_, *dfrA1*, *mcr-1.1*, *mdf*(A)-like, *sul1*, *sul2*, *tet*(A) | ERS2055644 |
| **U16-0042** | 2016 | turkey | ST-1564 | 2 | *Inc*FIC(FII), *Inc*I1, *Inc*FIB(AP001918), p0111, ***Inc*X4**, *Col*(MG828) | *astA, gad, iroN, iss, mchF, tsh* | *aadA1*-like, *bla*_TEM-1B_, *mcr-1.1*, *mdf*(A)-like, *qnrS1*, *sul2*, *tet*(A) | ERS2055671 |
| **U16-0047** | 2016 | turkey | ST-1170 | 2 | *Inc*FII, *Inc*FIB(AP001918), *Inc*X1, *Inc*Q1, ***Inc*X4** | *cma, gad, iroN, iss, vat* | *aadA1*, *aph(3’’)-Ib*, *aph(6)-Id*, *bla*_TEM-1B_, *dfrA1*, *mcr-1.1*, *mdf*(A)-like, *sul1*, *sul2*, *tet*(A) | ERS2055682 |
| **U16-0049** | 2016 | turkey | ST-1170 | 2 | *Inc*FII, *Inc*FIB(AP001918), *Inc*X1, *Inc*Q1, *Col*156, ***Inc*X4** | *cma, gad, iroN, iss, vat* | *aadA1*, *aph(3’’)-Ib*, *aph(6)-Id*, *bla*_TEM-1B_, *dfrA1*, *mcr-1.1*, *mdf*(A)-like, *sul1*, *sul2*, *tet*(A) | ERS2055681 |
| **U16-0063** | 2016 | turkey | ST-88 | 2 | *Inc*FIB(AP001918), *Inc*FIC(FII), *Inc*FII(pHN7A8), ***Inc*X4**, *Col*RNAI | *gad, iroN, iss, lpfA, mchF, mcmA* | *aadA2*4-like, *aadB*, *bla*_OXA-1_, *bla*_TEM-1B_, *catA1*-like, *floR*-like, *mcr-1.1*, *mdf*(A)-like, *sul1*, *sul2*, *tet*(B) | ERS2055679 |
| **U16-0081X** | 2016 | turkey | ST-69 | 4 | p0111, *Inc*X3, *Inc*X4, *Col*(MG828) | *air, eilA, gad, iss, lpfA* | *bla*_SHV-12_, *mcr-1.1*, *mdf*(A)-like, *qnrS1* | ERS2055667 |
| **U16-0083X** | 2016 | turkey | ST-48 | 2 | *Inc*HI1B(CIT), *Inc*I1, p0111, *Col*(MG828), ***Inc*X4**, *Col*RNAI | *gad* | *bla*_CMY-2_, *bla*_TEM-1B_, *mcr-1.1*, *mdf*(A)-like, *qnrB19*, *tet*(A)-like | ERS2055689 |
| **U16-0091** | 2016 | turkey | ST-349 | 4 | *Inc*FIB(AP001918), *Inc*FII(pCoo), p0111, ***Inc*X4**, *Col*(MG828) | *air, cma, eilA, iroN, iss* | *bla*_TEM-1B_-like, *mcr-1.1*, *mdf*(A)-like, *tet*(A)-like | ERS2055683 |
| **U16-0100** | 2016 | turkey | ST-359 | 4 | *Inc*FIC(FII), *Inc*FII, *Inc*I1, *Inc*FIB(AP001918), *Inc*Q1, ***Inc*X4**, *Col*RNAI | *cba, cma, gad, iroN, iss, lpfA* | *aadA1*, *aph(3’’)-Ib*, *aph(6)-Id*, *bla*_TEM-1B_, *dfrA1*, *mcr-1.1*, *mdf*(A)-like, *sul1*, *sul2*, *tet*(A) | ERS2055680 |
| **U16-0101** | 2016 | turkey | ST-919 | 2 | *Inc*FII, *Inc*FIB(AP001918), *Inc*N, p0111, ***Inc*X4**, *Col*(MG828) | *gad, iha, iroN, iss, vat* | *aph(3’’)-Ib*-like, *aph(6)-Id*, *bla*_TEM-1B_-like, *mcr-1.1*, *mdf*(A)-like, *tet*(A) | ERS2055677 |
| **U16-0109X** | 2016 | broiler | ST-398 | 2 | *Inc*HI2, *Inc*HI2A, *Inc*FII, *Inc*I1, *Inc*FIB(AP001918), *Inc*X1, TrfA, *Inc*B/O/K/Z, *Col*(MG828) | *gad, iroN, iss, mchF* | *aadA1*, *aph(3’)-Ia*-like, *bla*_CMY-2_, *bla*_TEM-1C_, *dfrA1*, *mcr-1.1*, *mdf*(A)-like, *sul2*, *tet*(A), *tet*(B)-like | ERS2055686 |
| **U16-0114** | 2016 | turkey | ST-2509 | 2 | *Inc*HI1B(CIT), *Inc*FII, *Inc*FIB(AP001918), *Inc*FIB(pLF82), p0111, *Inc*Q1, ***Inc*X4**, *Col*(MG828) | *gad, iroN, iss, lpfA* | *aadA1*, *aph(3’’)-Ib*, *aph(6)-Id*, *bla*_TEM-1B_, *dfrA1*, *floR*-like, *mcr-1.1*, *mdf*(A)-like, *sul1*, *sul2*-like, *tet*(A)-like | ERS2055674 |
| **# U16-0115** | 2016 | turkey | ST-354 | 4 | *Inc*HI2A, *Inc*FII, ***Inc*HI2**, *Inc*FIB(AP001918), TrfA, *Col*RNAI | *air, eilA, gad, iroN, iss, lpfA* | *aac(3)-IIa*, *aadA1*, *aadA2*, *aph(3’)-Ia*-like, *aph(3’’)-Ib*, *aph(6)-Id*, *bla*_TEM-1A_, *catA1*-like, *cmltA1*-like, *mcr-1.1*, *mdf*(A)-like, *sul3*, *tet*(A) | ERS2055668 |
| **# U16-0115X** |  |  | ST-359 | 2 | *Inc*FIB(AP001918), *Inc*I1, *Inc*FIC(FII), ***Inc*X4,** *Col*(MG828) | *gad, iroN, iss, lpfA, tsh* | *aac(3)-IId*-like, *aadA1*-like, *bla*_TEM-1B_, *mcr-1.1*, *mdf*(A)-like, *tet*(A) | ERS2055672 |
| **U16-0142X** | 2016 | broiler | ST-359 | 2 | *Inc*I1, p0111, ***Inc*X4**, *Col*RNAI | *gad, lpfA* | *aadA1*-like, *aad*A5-like, *bla*_SHV-12_, *bla*_TEM-1B_, *catA1*-like, *dfrA1*, *dfrA17*, *mcr-1.1*, *mdf*(A)-like, *sul1*, *tet*(A)-like | ERS2055642 |
| **U16-0149** | 2016 | turkey | ST-602 | 2 | *Inc*FII(pRSB107), *Inc*FIB(AP001918), *Inc*X1, ***Inc*X4**, *Col*(MG828) | *gad, iroN, iss, lpfA, mchF* | *bla*_TEM-1B_, *mcr-1.1*, *mdf*(A)-like, *qnrS1* | ERS2055678 |
| **U16-0251X** | 2016 | turkey | ST-117 | 4 | *Inc*FIB(pLF82), *Inc*FIB(AP001918), *Col*156, ***Inc*X4**, *Col*RNAI | *astA, cma, gad, iroN, iss, lpfA, vat* | *aadA1*, *aadA2*, *bla*_TEM-52C_, *cmltA1*-like, lnu(F), *mcr-1.1*, *mdf*(A)-like, *qnrB19*, *sul3* | ERS2055669 |
| **U16-0254** | 2016 | turkey | ST-2556 | 2 | *Inc*FIB(AP001918), *Inc*I1, *Inc*FIC(FII), *Col*8282, *Col*(MG828), *Col*156, ***Inc*X4**, *Col*RNAI | *cba, cma, iroN, iss, mchF* | *aac(3)-IId*-like, *aadA1*-like, *bla*_TEM-1B_, *mcr-1.1*, *mdf*(A)-like, *tet*(A) | ERS2055690 |
| **U16-0255X** | 2016 | turkey | ST-155 | 2 | *Inc*FII, *Inc*I1, *Inc*FIB(AP001918), *Col*(MG828), ***Inc*X4**, *Col*RNAI | *astA, cma, gad, iroN, iss, lpfA* | *bla*_CMY-2_, *bla*_TEM-1B_, *dfrA17*, *mcr-1.1, mdf*(A)-like | ERS2055645 |
| **U16-0258** | 2016 | turkey | ST-191 | 2 | *Inc*FIC(FII), *Inc*FIA, *Inc*I1, *Inc*FIB(AP001918), ***Inc*X4** | *gad, iroN, iss, lpfA, mchF, tsh* | *aadA1*, *bla*_TEM-1C_, *dfrA1*, *mcr-1.1*, *mdf*(A)-like, *sul1*, *tet*(A) | ERS2055694 |
| **U16-0259** | 2016 | turkey | ST-354 | 2 | *Inc*FIA, *Inc*FIB(AP001918), *Inc*FII(pCoo), *Inc*X1, *Col*(MG828) | *air, gad, lpfA* | *bla*_TEM-1B_, *mcr-1.1*, *mdf*(A)-like, *tet*(B), *lnu*(G) | ERS2055691 |
| **U16-0271** | 2016 | turkey | ST-48 | 2 | *Inc*FIA(HI1), *Inc*HI1B(R27), *Inc*HI1A, ***Inc*X4** | *gad* | *aac(3)-IIa*, *aadA1*-like, *aadA2*, *aph(3’)-Ia*-like, *aph(3’’)-Ib*, *aph(6)-Id*, *bla*_TEM-1B_, *cmltA1*-like, *mcr-1.1*, *mdf*(A)-like, *mph*(E), *msr*(E), *qnrB19*, *sul3*, *tet*(B) | ERS2055688 |
| **U16-0272** | 2016 | turkey | ST-227 | 4 | *Inc*HI2A, *Inc*FIC(FII), ***Inc*HI2**, *Inc*FIB(AP001918), TrfA | *capU, cba, cma, gad, iroN, iss, mchF, tsh* | *aadA1*, *aadA2*, *aph(3’’)-Ib*, *aph(6)-Id*, *bla*_TEM-1B_, *catA1*-like, *cmltA1*-like, *dfrA1*, *mcr-1.1*, *mdf*(A)-like, *sul1*, *sul3*, *tet*(A) | ERS2055692 |
| **U16-0279** | 2016 | broiler | ST-1303 | 2 | *Inc*HI1B(CIT), p0111, *Col*156, ***Inc*X4**, *Col*RNAI | *astA, gad* | *aph(3’’)-Ib*-like, *aph(6)-Id*, *bla*_TEM-1B_-like, *dfrA14*, *mcr-1.1*, *mdf*(A)-like, *qnrB19*, *sul2*, *tet*(A)-like | ERS2055640 |
| **U16-0288X** | 2016 | turkey | ST-354 | 2 | *Inc*HI2A, *Inc*FII(29), ***Inc*HI2**, *Inc*FIB(AP001918), *Inc*FII, TrfA, p0111, *Col*(MG828), *Col*RNAI | *air, astA, cba, cma, eilA, gad, iroN, iss, lpfA, mchF* | *aac(3)-IIa*, *aadA1*-like, *aadA2*, *aph(3’)-Ia*-like, *aph(3’’)-Ib*-like, *aph(6)-Id*, *bla*_TEM-1C_, *cmltA1*-like, *dfrA1*, *mcr-1.1*, *mdf*(A)-like, *sul1*, *sul3*, *tet*(A) | ERS2055641 |
| **U16-0303** | 2016 | turkey | ST-90 | 2 | *Inc*FII, *Inc*FIB(AP001918), *Inc*Q1, *Col*156, ***Inc*X4**, *Col*RNAI | *cma, gad, iroN, iss* | *aadA1*, *aph(3’’)-Ib*, *aph(6)-Id*, *bla*_TEM-1B_, *dfrA1*, *mcr-1.1*, *mdf*(A)-like, *sul1*, *sul2*, *tet*(A) | ERS2055684 |
| **U16-0307X** | 2016 | turkey | ST-58 | 2 | *Inc*FIB(pLF82), *Inc*FII, *Inc*I1, *Inc*FIC(FII), *Inc*FIB(AP001918), *Inc*X1, *Inc*R, ***Inc*X4** | *gad, lpfA* | *aadA1*, *aadA2*, *bla*_SHV-12_, *bla*_TEM-1B_, *cmltA1*-like, *dfrA1*2, *floR*-like, *mcr-1.1*, *sul3*, *tet*(A)-like, *tet*(B)-like, *tet*(M)-like | ERS2055687 |
| **## U16-0308** | 2016 | turkey | ST-10 | 4 | *Inc*HI1B(CIT), *Col*RNAI, *Col*(MG828), *Col*156, *Inc*X1, p0111, ***Inc*X4** | *gad* | *bla*_TEM-1B_, *mcr-1.1*, *mdf*(A)-like, *qnrS1*, *tet*(A)-like | ERS2055693 |
| **## U16-0308X** |  |  | ST-48 | 2 | *Inc*I1, p0111, *Col*(MG828), ***Inc*X4**, *Col*RNAI | *gad* | *bla*_CMY-2_, *bla*_TEM-1B_, *mcr-1.1*, *mdf*(A)-like, *qnrB19*, *tet*(A)-like | ERS2055636 |
| **U16-0311** | 2016 | turkey | ST-354 | 2 | *Inc*FII(pRSB107), *Inc*I1, *Inc*FIB(AP001918), ***Inc*X4** | *air, astA, cma, eilA, gad, iha, iroN, iss, lpfA* | *aadA1*, *aadA2*, *bla*_TEM-1B_, *cmltA1*-like, *mcr-1.1*, *mdf*(A)-like, *sul3*, *tet*(A) | ERS2055685 |
| **U16-0313** | 2016 | turkey | ST-6286 | 4 | *Inc*FIB(AP001918), *Inc*FII(29), *Inc*I1, *Inc*FIC(FII), *Col*(MG828), *Col*RNAI, *Col*156, p0111, ***Inc*X4**, *Col*8282 | *cma, ireA, iroN, iss, lpfA, pic* | *aadA1*, *aadA2*, *cmltA1*-like, *mcr-1.1*, *mdf*(A)-like, *qnrB19*, *sul3*, *tet*(A) | ERS2055670 |
| **U16-0323** | 2016 | turkey | ST-602 | 2 | *Inc*FII, *Inc*FIB(AP001918), *Inc*X1, ***Inc*X4** | *gad, iroN, iss, lpfA, mchF* | *bla*_TEM-1B_, *mcr-1.1*, *mdf*(A)-like, *qnrS1*, *aph(3’’)-Ib*, *aph(6)-Id*, *tet*(B) | ERS2055638 |
| **U16-0343** | 2016 | turkey | ST-1167 | 4 | *Inc*HI2A, *Inc*FII(29), *Inc*HI2, *Inc*FIB(AP001918), TrfA, *Col*(MG828), *Col*RNAI, *Col*156, *Inc*X1, ***Inc*X4** | *gad, iss, lpfA* | *aad*A5, *bla*_TEM-1B_, *dfrA17*, *mcr-1.1*, *mdf*(A)-like, *qnrB19*, *sul1*, *sul2*, *tet*(A)-like | ERS2055639 |
| **U16-0351** | 2016 | broiler | ST-86 | 2 | *Inc*FII, *Inc*FIB(AP001918), *Col*(MG828), *Inc*Q1, ***Inc*X4**, *Col*RNAI | *gad, iroN, iss, lpfA* | *aadA1*, *aph(3’’)-Ib*, *aph(6)-Id*, *bla*_TEM-1B_-like, *dfrA1*, *floR*, *mcr-1.1*, *mdf*(A)-like, mph(B), *sul1*, *sul2*, *tet*(A) | ERS2055637 |
| **U16-0523** | 2016 | broiler | ST-37 | 4 | *Inc*FII, *Inc*FIB(AP001918), *Inc*X1, ***Inc*X4**, *Col*(MG828) | *gad, iroN, iss* | *aadA1*, *bla*_TEM-1B_-like, *dfrA1*, *mcr-1.1*, *mdf*(A)-like, *tet*(A) | ERS2055631 |
| **U16-0565X** | 2016 | turkey | ST-3897 | 2 | *Inc*HI2A, ***Inc*HI2**, *Inc*FIB(AP001918), *Inc*I1, TrfA, *Col*RNAI | *astA, cma, gad, iroN, iss* | *aadA1*, *aadA2*, *aph(3’’)-Ib*, *aph(6)-Id*, *bla*_TEM-1B_,  *catA1*-like, *cmltA1*-like, *dfrA1*, *lnu*(F), *mcr-1.1*, *mdf*(A)-like, *sul1*, *sul3*, *tet*(A) | ERS2055633 |
| **U16-0575X** | 2016 | turkey | ST-3897 | 2 | *Inc*HI2A, *Inc*HI2, *Inc*FIB(AP001918), *Inc*I1, TrfA, *Col*RNAI | *astA, cma, gad, iroN, iss* | *aph(3’’)-Ib*, *aph(6)-Id*, *bla*_TEM-1B_, *catA1*-like, *cmltA1*-like, *dfrA1*, *lnu*(F), *mcr-1.1*, *mdf*(A)-like, *sul1*, *sul2*-like, *sul3*, *tet*(A) | ERS2055634 |
| **U16-0661X** | 2016 | turkey | ST-1611 | 2 | *Inc*FIC(FII), *Inc*HI1B(CIT), *Inc*FIA, *Inc*I1, *Inc*FIB(AP001918), *Col*(BS512), *Inc*X1, p0111, ***Inc*X4** | *gad, iroN, iss, lpfA, mchF, tsh* | *aph(3’’)-Ib*-like, *aph(6)-Id*, *bla*_CTX-M-1_, *bla*_TEM-1B_, *dfrA14*, *mcr-1.1*, *mdf*(A)-like, *qnrS1*, *sul2*, *tet*(A)-like | ERS2055635 |
| **U16-0741X** | 2016 | broiler | ST-1011 | 2 | *Inc*FII, *Inc*I1, *Inc*FIB(AP001918), *Inc*N, *Inc*Q1, ***Inc*X4** | *air, astA, cma, eilA, iroN, iss* | *aadA1*, *aph(3’’)-Ib*, *aph(6)-Id, bla*_SHV-12_, *bla*_TEM-1B_, *dfrA1*, *mcr-1.1*, *mdf*(A)-like, *sul1*, *sul2*, *tet*(A) | ERS2055632 |
