# Supplementary material for: Occurrence and Characterization of mcr-1-Positive Escherichia coli Isolated From Food-Producing Animals in Poland, 2011–2016
Source: Front Microbiol. 2019 Aug 8;10:1753. doi: 10.3389/fmicb.2019.01753 (PMC6694793; doi:10.3389/fmicb.2019.01753)
Supplement: Supplementary file 2 [file Table_2.DOCX]

Supplementary Material

Supplementary Table S2. Detailed information about source of isolates. nd- not defined

| **Sample name** | **Isolation year** | **Isolation source** | **ID slaughterhouse** | **ID farm** | **Animal age (weeks)** | **Number of animals in flock/herd** |
| --- | --- | --- | --- | --- | --- | --- |
| **P-12-17510** | 2012 | turkey | S1 | F1 | nd | nd |
| **U16-0575X** | 2016 | turkey | S1 | F1 | 16 | 19500 |
| **U16_0579** | 2016 | broiler | S2 | F2 | 6 | 4497 |
| **11-09701** | 2011 | turkey | S3 | F3 | 21 | 12500 |
| **P-12-00505** | 2012 | turkey | S3 | F3 | 21 | 9200 |
| **P-12-12952** | 2012 | turkey | S3 | F3 | 15 | 15000 |
| **P-13-19081** | 2013 | turkey | S3 | F3 | 19 | 16700 |
| **U14_0076** | 2014 | turkey | S3 | F3 | 15 | 4900 |
| **U14-0397** | 2014 | turkey | S3 | F3 | 18 | 8500 |
| **P-12-16000** | 2012 | turkey | S4 | F4 | 20 | 6200 |
| **U14_0930** | 2014 | turkey | S4 | F4 | 16 | 6200 |
| **U14_1004** | 2014 | turkey | S4 | F4 | 20 | 4300 |
| **U14_1070** | 2014 | turkey | S4 | F4 | 20 | 4700 |
| **U14_1100_1** | 2014 | turkey | S4 | F4 | 16 | 7800 |
| **U14_1100_3** | 2014 | turkey | S4 | F4 | 16 | 7800 |
| **U14_1192** | 2014 | turkey | S4 | F4 | 23 | 1750 |
| **U14-0034** | 2014 | turkey | S4 | F4 | 16 | 8000 |
| **U16-0149** | 2016 | turkey | S4 | F4 | 16 | 5800 |
| **U16-0015** | 2016 | broiler | S5 | F5 | 6 | 24000 |
| **U16-0015X** | 2016 | broiler | S5 | F5 | 6 | 24000 |
| **U16-0741X** | 2016 | broiler | S5 | F5 | 6 | 67300 |
| **U14_0002** | 2014 | broiler | S6 | F5 | 5 | 72000 |
| **U14-0628** | 2014 | turkey | S6 | F6 | 14 | 950 |
| **U16-0351** | 2016 | broiler | S7 | F7 | 4 | 50000 |
| **U16-0303** | 2016 | turkey | S8 | F8 | 15 | 5000 |
| **U16-0063** | 2016 | turkey | S9 | F9 | 15 | 3400 |
| **U16-0279** | 2016 | broiler | S9 | F9 | 6 | 60000 |
| **U16-0114** | 2016 | turkey | S10 | F10 | 16 | 2000 |
| **U16-0115** | 2016 | turkey | S10 | F10 | 16 | 6000 |
| **U16-0115X** | 2016 | turkey | S10 | F10 | 16 | 6000 |
| **U16-0109X** | 2016 | broiler | S11 | F11 | 6 | 100299 |
| **U16-0271** | 2016 | turkey | S11 | F11 | 12 | 6500 |
| **U16-0272** | 2016 | turkey | S11 | F11 | 12 | 22500 |
| **U16-0323** | 2016 | turkey | S11 | F11 | 12 | 6500 |
| **U16-0565X** | 2016 | turkey | S11 | F11 | 13 | 19500 |
| **E14_0154B** | 2014 | cattle | S12 | F12 | 2 | 30 |
| **U16-0288X** | 2016 | turkey | S13 | F13 | 15 | 2000 |
| **P-12-16114** | 2012 | turkey | S14 | F14 | 20 | 2000 |
| **P-12-10660** | 2013 | turkey | S14 | F14 | 16 | 6000 |
| **P-12-06286** | 2012 | broiler | S15 | F15 | 6 | 39000 |
| **U14_0022** | 2014 | turkey | S16 | F16 | 16 | 15000 |
| **U14_0138** | 2014 | turkey | S16 | F16 | 19 | 3000 |
| **U16-0251X** | 2016 | turkey | S16 | F16 | 16 | 4500 |
| **U16-0313** | 2016 | turkey | S16 | F16 | 20 | 2500 |
| **P-12-12187** | 2013 | laying hen | S17 | F17 | 52 | 25000 |
| **P-12-12188** | 2013 | laying hen | S17 | F17 | 48 | 25000 |
| **E14_0351A** | 2014 | broiler | S17 | F17 | 5 | 8100 |
| **U16-0259** | 2016 | turkey | S18 | F18 | 16 | 7500 |
| **U16-0311** | 2016 | turkey | S18 | F18 | 15 | 12000 |
| **P-12-01553** | 2013 | turkey | S19 | F19 | 20 | 2040 |
| **U16-0047** | 2016 | turkey | S19 | F19 | 16 | 1836 |
| **U16-0049** | 2016 | turkey | S19 | F19 | 21 | 2200 |
| **U16-0100** | 2016 | turkey | S19 | F19 | 21 | 4100 |
| **U16-0101** | 2016 | turkey | S19 | F19 | 20 | 1010 |
| **U16-0254** | 2016 | turkey | S19 | F19 | 20 | 4100 |
| **U16-0041X** | 2016 | turkey | S20 | F20 | 16 | 1400 |
| **U16-0042** | 2016 | turkey | S20 | F20 | 21 | 900 |
| **U16-0091** | 2016 | turkey | S20 | F20 | 21 | 1650 |
| **U16-0255X** | 2016 | turkey | S20 | F20 | 16 | 1980 |
| **U16-0258** | 2016 | turkey | S20 | F20 | 16 | 1980 |
| **U16-0307X** | 2016 | turkey | S20 | F20 | 16 | 2000 |
| **U16-0308** | 2016 | turkey | S20 | F20 | 21 | 1080 |
| **U16-0308X** | 2016 | turkey | S20 | F20 | 21 | 1080 |
| **U14_0810** | 2014 | turkey | S21 | F21 | 15 | 1728 |
| **11-14155** | 2011 | turkey | S22 | F23 | 16 | 11000 |
| **U14_0418** | 2014 | turkey | S22 | F23 | 15 | 120000 |
| **U14_0692** | 2014 | turkey | S22 | F23 | 15 | 11000 |
| **U14_0857** | 2014 | turkey | S22 | F23 | 20 | 3000 |
| **P-12-00759** | 2012 | turkey | S23 | F22 | 21 | 1724 |
| **U14_0089** | 2014 | turkey | S23 | F22 | 21 | 4100 |
| **U14_0306** | 2014 | turkey | S23 | F22 | 21 | 7600 |
| **U16-0016X** | 2016 | turkey | S23 | F22 | 21 | 2960 |
| **U16-0024** | 2016 | turkey | S23 | F22 | 16 | 3297 |
| **U16-0081X** | 2016 | turkey | S23 | F22 | 16 | 3289 |
| **U16-0083X** | 2016 | turkey | S23 | F22 | 16 | 3950 |
| **U16-0142X** | 2016 | broiler | S24 | F25 | 6 | 24000 |
| **U16-0523** | 2016 | broiler | S24 | F24 | 5 | 5000 |
| **U16-0343** | 2016 | turkey | S25 | F25 | 18 | 10000 |
| **U16-0661X** | 2016 | turkey | S26 | F26 | 19 | 6300 |
| **U15_0035X** | 2015 | pig | S27 | F27 | 24 | 100 |
